# Supplementary material for: Soil fauna-microbial interactions shifts fungal and bacterial communities under a contamination disturbance
Source: PLoS One. 2023 Oct 25;18(10):e0292227. doi: 10.1371/journal.pone.0292227 (PMC10599570; doi:10.1371/journal.pone.0292227)
Supplement: S1 Table. A — B. Count reports for generated ASV. (ZIP) [file pone.0292227.s001.zip › TableS1B.docx]

|  | **16S**  **rRNA** | **ITS**  **region** | **PAH RHD GN** | **PAH RHD GP** |
| --- | --- | --- | --- | --- |
| **total_reads** | 16,172,736 | 23,079,526 | 31,702,674 | 25,928,318 |
| **contaminants_reads** | 42,614 | 34,708 | 11,408,476 | 999,512 |
| **phix_reads** | 706 | 630 | 28,342 | 4,856 |
| **non_contam_non_phix_reads** | 16,129,614 | 23,044,188 | 20,265,856 | 24,923,950 |
| **non_contam_non_phix_reads_1** | 8,064,807 | 11,522,093 | 10,132,928 | 12,461,975 |
| **assembled_reads_QC_passed** | 7,795,754 | 6,946,010 | 6,715,225 | 4,165,994 |

**Table S1B.** Read counts report for whole *Bacteria*, *Archaea* and *Fungi* communities and for PAH Gram-negative (GN) and Gram-positive (GP) bacterial degraders (ASV).
