# Supplementary material for: Soil fauna-microbial interactions shifts fungal and bacterial communities under a contamination disturbance
Source: PLoS One. 2023 Oct 25;18(10):e0292227. doi: 10.1371/journal.pone.0292227 (PMC10599570; doi:10.1371/journal.pone.0292227)
Supplement: S3 Table — (DOCX) [file pone.0292227.s003.docx]

**Table S3.** Median Shannon H’ diversity and median ASV. Legend as described in **Table 1**.

|  | **ITS** | | **16S rRNA** | | **PAH - RHD Gram Negative** | | **PAH - RHD Gram Positive** | |
| --- | --- | --- | --- | --- | --- | --- | --- | --- |
|  | **gene** | | **gene** | | **gene** | | **gene** | |
|  | **median ± MAD (n=6)** | | **median ± MAD (n=6)** | | **median ± MAD (n=6)** | | **median ± MAD (n=6)** | |
|  | **Shannon** | **ASV n^o^** | **Shannon** | **ASV n^o^** | **Shannon** | **ASV n^o^** | **Shannon** | **ASV n^o^** |
| **BF bulk CTRL** | 1.48 ± 1.613 | 75 ± 69.682 | 5.045 ± 0.149 | 463 ± 87.473 | 2.766 ± 1.353 | 29.5 ± 30.393 | 4.944 ± 0.335 | 227 ± 92.662 |
| **BF bulk PHE** | 0.746 ± 0.73 | 53.5 ± 51.15 | 5.446 ± 0.16 | 473.5 ± 83.026 | 3.36 ± 0.226 | 40.5 ± 6.672 | 5.127 ± 0.153 | 242 ± 54.856 |
| **BF rhizosphere CTRL** | 1.617 ± 1.727 | 108 ± 44.478 | 5.35 ± 0.376 | 535 ± 111.936 | 3.496 ± 0.069 | 51 ± 9.637 | 5.108 ± 0.401 | 281.5 ± 85.25 |
| **BF rhizosphere PHE** | 1.643 ± 1.307 | 136.5 ± 112.678 | 5.215 ± 0.129 | 498 ± 122.314 | 3.349 ± 0.103 | 57.5 ± 16.309 | 5.572 ± 0.049 | 409.5 ± 34.1 |
| **C bulk CTRL** | 1.233 ± 0.441 | 61.5 ± 3.706 | 4.723 ± 0.317 | 342.5 ± 78.578 | 2.675 ± 0.091 | 17.5 ± 2.965 | 4.674 ± 0.208 | 137 ± 11.119 |
| **C bulk PHE** | 0.723 ± 0.186 | 34.5 ± 13.343 | 5.118 ± 0.39 | 371.5 ± 103.782 | 2.997 ± 0.205 | 34 ± 7.413 | 5.017 ± 0.056 | 209 ± 27.428 |
| **C rhizosphere CTRL** | 1.255 ± 0.816 | 97 ± 37.806 | 5.189 ± 0.151 | 455.5 ± 25.204 | 3.267 ± 0.405 | 37 ± 13.343 | 5.242 ± 0.562 | 281 ± 64.493 |
| **C rhizosphere PHE** | 1.101 ± 0.522 | 87 ± 40.03 | 5.312 ± 0.201 | 477.5 ± 50.408 | 3.497 ± 0.355 | 50 ± 15.567 | 5.585 ± 0.123 | 409.5 ± 88.956 |
| **CE bulk CTRL** | 1.17 ± 1.064 | 63 ± 47.443 | 4.99 ± 0.321 | 405.5 ± 107.488 | 3.037 ± 0.394 | 25 ± 2.965 | 4.966 ± 0.125 | 211.5 ± 25.204 |
| **CE bulk PHE** | 0.835 ± 0.504 | 33 ± 18.532 | 5.311 ± 0.106 | 429 ± 106.006 | 3.063 ± 0.551 | 31.5 ± 2.965 | 5.479 ± 0.183 | 328 ± 92.662 |
| **CE rhizosphere CTRL** | 2.093 ± 0.977 | 104 ± 52.632 | 5.258 ± 0.453 | 472.5 ± 161.603 | 2.88 ± 0.392 | 27.5 ± 14.085 | 5.038 ± 0.678 | 263 ± 122.314 |
| **CE rhizosphere PHE** | 1.262 ± 1.103 | 108 ± 59.304 | 5.428 ± 0.129 | 552 ± 44.478 | 3.472 ± 0.117 | 55 ± 4.448 | 5.584 ± 0.137 | 432.5 ± 29.652 |
| **CEN bulk CTRL** | 2.102 ± 0.898 | 82 ± 47.443 | 4.92 ± 0.615 | 337 ± 85.25 | 2.581 ± 0.072 | 20 ± 6.672 | 4.854 ± 0.369 | 189.5 ± 66.717 |
| **CEN bulk PHE** | 0.944 ± 0.375 | 50 ± 45.961 | 5.411 ± 0.092 | 473 ± 80.802 | 3.58 ± 0.305 | 52.5 ± 5.189 | 5.197 ± 0.071 | 225 ± 22.98 |
| **CEN rhizosphere CTRL** | 2.835 ± 1.799 | 153 ± 34.1 | 5.175 ± 0.265 | 482 ± 48.926 | 3.05 ± 0.631 | 35.5 ± 23.722 | 5.038 ± 0.416 | 219 ± 146.777 |
| **CEN rhizosphere PHE** | 1.435 ± 0.409 | 91 ± 32.617 | 5.304 ± 0.244 | 557 ± 37.065 | 3.499 ± 0.307 | 49.5 ± 5.93 | 5.607 ± 0.152 | 410 ± 29.652 |
| **CN bulk CTRL** | 0.828 ± 0.78 | 40 ± 11.119 | 4.91 ± 0.265 | 337 ± 76.354 | 2.911 ± 0.292 | 33.5 ± 10.378 | 4.692 ± 0.121 | 149.5 ± 25.204 |
| **CN bulk PHE** | 0.658 ± 0.228 | 28.5 ± 7.413 | 4.914 ± 0.161 | 315.5 ± 40.03 | 3.178 ± 0.327 | 32.5 ± 2.224 | 5.233 ± 0.061 | 243.5 ± 25.204 |
| **CN rhizosphere CTRL** | 1.225 ± 0.7 | 113.5 ± 40.771 | 5.35 ± 0.251 | 462 ± 48.184 | 2.979 ± 0.648 | 30.5 ± 14.085 | 5.37 ± 0.182 | 355 ± 131.21 |
| **CN rhizosphere PHE** | 0.843 ± 0.198 | 98.5 ± 30.393 | 5.619 ± 0.165 | 571.5 ± 66.717 | 3.394 ± 0.285 | 44 ± 15.567 | 5.68 ± 0.162 | 457 ± 42.254 |
| **E bulk CTRL** | 1.056 ± 0.812 | 75.5 ± 54.856 | 5.207 ± 0.25 | 418.5 ± 110.454 | 2.588 ± 0.051 | 22.5 ± 7.413 | 4.781 ± 0.134 | 158 ± 40.771 |
| **E bulk PHE** | 0.669 ± 0.528 | 39.5 ± 32.617 | 5.33 ± 0.299 | 477 ± 144.553 | 3.447 ± 0.299 | 52 ± 17.05 | 5.163 ± 0.116 | 230 ± 26.687 |
| **E rhizosphere CTRL** | 2.41 ± 0.698 | 120 ± 25.945 | 5.222 ± 0.344 | 522 ± 42.254 | 3.264 ± 1.061 | 46.5 ± 39.289 | 4.933 ± 0.352 | 185 ± 43.737 |
| **E rhizosphere PHE** | 0.793 ± 0.249 | 105 ± 28.169 | 5.338 ± 0.138 | 481 ± 86.732 | 3.387 ± 0.476 | 41 ± 10.378 | 5.519 ± 0.149 | 360 ± 81.543 |
| **EN bulk CTRL** | 0.949 ± 0.991 | 49 ± 28.911 | 5.221 ± 0.119 | 461 ± 25.204 | 2.573 ± 1.205 | 19 ± 23.722 | 4.811 ± 0.472 | 162.5 ± 104.523 |
| **EN bulk PHE** | 0.53 ± 0.169 | 25.5 ± 16.309 | 5.323 ± 0.455 | 458 ± 201.634 | 3.404 ± 0.458 | 55.5 ± 16.309 | 5.135 ± 0.178 | 209 ± 20.015 |
| **EN rhizosphere CTRL** | 0.894 ± 0.846 | 79 ± 50.408 | 5.225 ± 0.273 | 523.5 ± 36.324 | 3.376 ± 0.415 | 45 ± 23.722 | 5.053 ± 0.536 | 221.5 ± 89.697 |
| **EN rhizosphere PHE** | 0.841 ± 0.373 | 98 ± 41.513 | 5.533 ± 0.321 | 574.5 ± 154.932 | 3.408 ± 0.256 | 48 ± 8.154 | 5.659 ± 0.055 | 430 ± 11.861 |
| **N bulk CTRL** | 2.428 ± 1.466 | 91 ± 57.821 | 5.037 ± 0.108 | 377 ± 83.767 | 2.727 ± 0.19 | 20 ± 8.154 | 4.758 ± 0.332 | 164 ± 35.582 |
| **N bulk PHE** | 0.555 ± 0.371 | 29.5 ± 17.791 | 5.391 ± 0.162 | 502 ± 98.593 | 3.121 ± 0.299 | 38.5 ± 8.896 | 5.158 ± 0.212 | 249 ± 48.926 |
| **N rhizosphere CTRL** | 1.594 ± 1.41 | 162 ± 89.697 | 5.211 ± 0.253 | 495.5 ± 51.891 | 3.276 ± 0.55 | 46 ± 29.652 | 5.33 ± 0.141 | 301.5 ± 62.269 |
| **N rhizosphere PHE** | 1.557 ± 1.464 | 153.5 ± 51.891 | 5.463 ± 0.324 | 533.5 ± 135.658 | 3.484 ± 0.26 | 54.5 ± 20.756 | 5.633 ± 0.114 | 436 ± 101.558 |
