# Supplementary material for: Soil fauna-microbial interactions shifts fungal and bacterial communities under a contamination disturbance
Source: PLoS One. 2023 Oct 25;18(10):e0292227. doi: 10.1371/journal.pone.0292227 (PMC10599570; doi:10.1371/journal.pone.0292227)
Supplement: S6 Table — (DOCX) [file pone.0292227.s006.docx]

**Table S6.** Summary of the three-way analysis of the variance (ANOVA) on the relative abundance of the ITS region ASVs identified at the genus level of *Fungi^a^*. ﻿

| **ANOVA tests results** | | | | | | |
| --- | --- | --- | --- | --- | --- | --- |
| ***Alternaria*** | **Df** | **Sum Sq** | **Mean Sq** | **F value** | **Pr(>F)** |  |
| **contamination** | **1** | **0.002** | **0.002** | **10.454** | **0.001** | ****** |
| compartment | 1 | 0.000 | 0.000 | 0.826 | 0.365 |  |
| SFMIC | 7 | 0.002 | 0.000 | 1.447 | 0.190 |  |
| contamination:compartment | 1 | 0.000 | 0.000 | 1.973 | 0.162 |  |
| **contamination:SFMIC** | **7** | **0.002** | **0.000** | **1.765** | **0.098** | **.** |
| compartment:SFMIC | 7 | 0.001 | 0.000 | 0.603 | 0.752 |  |
| contamination:compartment:SFMIC | 7 | 0.001 | 0.000 | 0.518 | 0.820 |  |
| Residuals | 159 | 0.030 | 0.000 |  |  |  |
| ***Chaetomium*** | **Df** | **Sum Sq** | **Mean Sq** | **F value** | **Pr(>F)** |  |
| contamination | 1 | 0.013 | 0.012 | 1.156 | 0.284 |  |
| compartment | 1 | 0.008 | 0.008 | 0.759 | 0.385 |  |
| **SFMIC** | **7** | **0.316** | **0.045** | **4.184** | **<0.001** | ******* |
| contamination:compartment | 1 | 0.017 | 0.017 | 1.595 | 0.209 |  |
| contamination:SFMIC | 7 | 0.112 | 0.016 | 1.490 | 0.174 |  |
| **compartment:SFMIC** | **7** | **0.133** | **0.019** | **1.765** | **0.098** | **.** |
| contamination:compartment:SFMIC | 7 | 0.024 | 0.003 | 0.315 | 0.946 |  |
| Residuals | 159 | 1.714 | 0.011 |  |  |  |
| ***Cladosporium*** | **Df** | **Sum Sq** | **Mean Sq** | **F value** | **Pr(>F)** |  |
| **contamination** | **1** | **0.010** | **0.010** | **5.710** | **0.018** | ***** |
| compartment | 1 | 0.002 | 0.002 | 1.122 | 0.291 |  |
| SFMIC | 7 | 0.020 | 0.003 | 1.694 | 0.114 |  |
| **contamination:compartment** | **1** | **0.006** | **0.006** | **3.304** | **0.071** | **.** |
| contamination:SFMIC | 7 | 0.004 | 0.001 | 0.366 | 0.921 |  |
| compartment:SFMIC | 7 | 0.004 | 0.001 | 0.378 | 0.914 |  |
| contamination:compartment:SFMIC | 7 | 0.008 | 0.001 | 0.677 | 0.691 |  |
| Residuals | 159 | 0.270 | 0.002 |  |  |  |
| ***Cystobasidium*** | **Df** | **Sum Sq** | **Mean Sq** | **F value** | **Pr(>F)** |  |
| **contamination** | **1** | **0.003** | **0.003** | **5.551** | **0.020** | ***** |
| **compartment** | **1** | **0.004** | **0.004** | **7.331** | **0.008** | ****** |
| **SFMIC** | **7** | **0.007** | **0.001** | **1.764** | **0.098** | **.** |
| contamination:compartment | 1 | 0.001 | 0.001 | 2.250 | 0.136 |  |
| contamination:SFMIC | 7 | 0.007 | 0.001 | 1.711 | 0.110 |  |
| compartment:SFMIC | 7 | 0.005 | 0.001 | 1.203 | 0.304 |  |
| contamination:compartment:SFMIC | 7 | 0.004 | 0.001 | 1.023 | 0.417 |  |
| Residuals | 159 | 0.087 | 0.001 |  |  |  |
| ***Gibberella*** | **Df** | **Sum Sq** | **Mean Sq** | **F value** | **Pr(>F)** |  |
| **contamination** | **1** | **0.016** | **0.016** | **3.777** | **0.054** | **.** |
| compartment | 1 | 0.003 | 0.003 | 0.682 | 0.410 |  |
| SFMIC | 7 | 0.043 | 0.006 | 1.428 | 0.198 |  |
| contamination:compartment | 1 | 0.000 | 0.000 | 0.012 | 0.911 |  |
| contamination:SFMIC | 7 | 0.035 | 0.005 | 1.171 | 0.323 |  |
| compartment:SFMIC | 7 | 0.018 | 0.003 | 0.604 | 0.752 |  |
| contamination:compartment:SFMIC | 7 | 0.033 | 0.005 | 1.084 | 0.376 |  |
| Residuals | 159 | 0.681 | 0.004 |  |  |  |
| ***Mortierella*** | **Df** | **Sum Sq** | **Mean Sq** | **F value** | **Pr(>F)** |  |
| **contamination** | **1** | **0.001** | **0.001** | **7.841** | **0.006** | ****** |
| **compartment** | **1** | **0.002** | **0.002** | **11.637** | **<0.001** | ******* |
| SFMIC | 7 | 0.002 | 0.000 | 1.585 | 0.143 |  |
| contamination:compartment | 1 | 0.000 | 0.000 | 2.183 | 0.142 |  |
| contamination:SFMIC | 7 | 0.001 | 0.000 | 0.787 | 0.599 |  |
| compartment:SFMIC | 7 | 0.001 | 0.000 | 0.771 | 0.612 |  |
| contamination:compartment:SFMIC | 7 | 0.001 | 0.000 | 0.672 | 0.696 |  |
| Residuals | 159 | 0.024 | 0.000 |  |  |  |
| ***Other Ascomycota*** | **Df** | **Sum Sq** | **Mean Sq** | **F value** | **Pr(>F)** |  |
| **contamination** | **1** | **0.008** | **0.008** | **5.626** | **0.019** | ***** |
| **compartment** | **1** | **0.016** | **0.016** | **10.480** | **0.001** | ****** |
| **SFMIC** | **7** | **0.019** | **0.003** | **1.822** | **0.086** | **.** |
| contamination:compartment | 1 | 0.000 | 0.000 | 0.001 | 0.978 |  |
| contamination:SFMIC | 7 | 0.009 | 0.001 | 0.851 | 0.547 |  |
| compartment:SFMIC | 7 | 0.006 | 0.001 | 0.553 | 0.793 |  |
| contamination:compartment:SFMIC | 7 | 0.009 | 0.001 | 0.828 | 0.566 |  |
| Residuals | 159 | 0.237 | 0.001 |  |  |  |
| ***Other Fungi*** | **Df** | **Sum Sq** | **Mean Sq** | **F value** | **Pr(>F)** |  |
| **contamination** | **1** | **0.019** | **0.019** | **8.480** | **0.004** | ****** |
| **compartment** | **1** | **0.025** | **0.025** | **11.395** | **<0.001** | ******* |
| **SFMIC** | **7** | **0.031** | **0.004** | **2.014** | **0.056** | **.** |
| contamination:compartment | 1 | 0.004 | 0.004 | 1.791 | 0.183 |  |
| contamination:SFMIC | 7 | 0.005 | 0.001 | 0.328 | 0.940 |  |
| compartment:SFMIC | 7 | 0.009 | 0.001 | 0.591 | 0.763 |  |
| contamination:compartment:SFMIC | 7 | 0.010 | 0.001 | 0.651 | 0.713 |  |
| Residuals | 159 | 0.348 | 0.002 |  |  |  |
| ***Other Ostropales*** | **Df** | **Sum Sq** | **Mean Sq** | **F value** | **Pr(>F)** |  |
| **contamination** | **1** | **0.008** | **0.008** | **10.862** | **0.001** | ****** |
| compartment | 1 | 0.000 | 0.000 | 0.307 | 0.580 |  |
| SFMIC | 7 | 0.005 | 0.001 | 0.918 | 0.494 |  |
| contamination:compartment | 1 | 0.000 | 0.000 | 0.549 | 0.460 |  |
| contamination:SFMIC | 7 | 0.002 | 0.000 | 0.501 | 0.833 |  |
| compartment:SFMIC | 7 | 0.004 | 0.001 | 0.868 | 0.533 |  |
| contamination:compartment:SFMIC | 7 | 0.004 | 0.001 | 0.902 | 0.507 |  |
| Residuals | 159 | 0.113 | 0.001 |  |  |  |
| ***Platygramme*** | **Df** | **Sum Sq** | **Mean Sq** | **F value** | **Pr(>F)** |  |
| contamination | 1 | 0.003 | 0.003 | 0.207 | 0.649 |  |
| compartment | 1 | 0.000 | 0.000 | 0.019 | 0.891 |  |
| SFMIC | 7 | 0.124 | 0.018 | 1.445 | 0.191 |  |
| contamination:compartment | 1 | 0.019 | 0.019 | 1.577 | 0.211 |  |
| contamination:SFMIC | 7 | 0.087 | 0.012 | 1.019 | 0.420 |  |
| compartment:SFMIC | 7 | 0.064 | 0.009 | 0.750 | 0.630 |  |
| contamination:compartment:SFMIC | 7 | 0.010 | 0.001 | 0.115 | 0.997 |  |
| Residuals | 159 | 1.941 | 0.012 |  |  |  |
| ***Pseudofabraea*** | **Df** | **Sum Sq** | **Mean Sq** | **F value** | **Pr(>F)** |  |
| contamination | 1 | 0.005 | 0.005 | 1.835 | 0.177 |  |
| compartment | 1 | 0.003 | 0.003 | 1.207 | 0.274 |  |
| SFMIC | 7 | 0.020 | 0.003 | 1.093 | 0.370 |  |
| contamination:compartment | 1 | 0.003 | 0.002 | 0.962 | 0.328 |  |
| contamination:SFMIC | 7 | 0.021 | 0.003 | 1.130 | 0.347 |  |
| compartment:SFMIC | 7 | 0.015 | 0.002 | 0.836 | 0.559 |  |
| contamination:compartment:SFMIC | 7 | 0.015 | 0.002 | 0.831 | 0.563 |  |
| Residuals | 159 | 0.413 | 0.003 |  |  |  |
| ***Rhodotorula*** | **Df** | **Sum Sq** | **Mean Sq** | **F value** | **Pr(>F)** |  |
| **contamination** | **1** | **0.003** | **0.003** | **5.631** | **0.019** | ***** |
| **compartment** | **1** | **0.002** | **0.002** | **4.312** | **0.039** | ***** |
| SFMIC | 7 | 0.004 | 0.001 | 1.285 | 0.261 |  |
| **contamination:compartment** | **1** | **0.002** | **0.002** | **4.379** | **0.038** | ***** |
| contamination:SFMIC | 7 | 0.004 | 0.001 | 1.115 | 0.356 |  |
| compartment:SFMIC | 7 | 0.003 | 0.000 | 1.048 | 0.400 |  |
| contamination:compartment:SFMIC | 7 | 0.003 | 0.000 | 0.851 | 0.547 |  |
| Residuals | 159 | 0.072 | 0.000 |  |  |  |
| ***Sphaerosporella*** | **Df** | **Sum Sq** | **Mean Sq** | **F value** | **Pr(>F)** |  |
| **contamination** | **1** | **2.403** | **2.403** | **23.075** | **<0.001** | ******* |
| compartment | 1 | 0.045 | 0.045 | 0.436 | 0.510 |  |
| **SFMIC** | **7** | **1.892** | **0.270** | **2.595** | **0.015** | ***** |
| contamination:compartment | 1 | 0.235 | 0.235 | 2.254 | 0.135 |  |
| contamination:SFMIC | 7 | 0.495 | 0.071 | 0.679 | 0.690 |  |
| compartment:SFMIC | 7 | 0.170 | 0.024 | 0.234 | 0.977 |  |
| contamination:compartment:SFMIC | 7 | 0.160 | 0.023 | 0.220 | 0.980 |  |
| Residuals | 159 | 16.557 | 0.104 |  |  |  |
| ***Zopfiella*** | **Df** | **Sum Sq** | **Mean Sq** | **F value** | **Pr(>F)** |  |
| **contamination** | **1** | **0.044** | **0.044** | **3.905** | **0.050** | ***** |
| compartment | 1 | 0.006 | 0.006 | 0.538 | 0.464 |  |
| **SFMIC** | **7** | **0.229** | **0.033** | **2.936** | **0.006** | ****** |
| contamination:compartment | 1 | 0.011 | 0.011 | 1.009 | 0.317 |  |
| contamination:SFMIC | 7 | 0.085 | 0.012 | 1.093 | 0.371 |  |
| compartment:SFMIC | 7 | 0.010 | 0.001 | 0.126 | 0.996 |  |
| contamination:compartment:SFMIC | 7 | 0.017 | 0.002 | 0.213 | 0.982 |  |
| Residuals | 159 | 1.773 | 0.011 |  |  |  |

^a^ Values in bold indicate significant or marginally significant effects. Df, degrees of freedom; F, variance ratio; Pr(>F), P value.
