# Supplementary material for: Soil fauna-microbial interactions shifts fungal and bacterial communities under a contamination disturbance
Source: PLoS One. 2023 Oct 25;18(10):e0292227. doi: 10.1371/journal.pone.0292227 (PMC10599570; doi:10.1371/journal.pone.0292227)
Supplement: S7 Table — (DOCX) [file pone.0292227.s007.docx]

**Table S7.** Summary of the three-way analysis of the variance (ANOVA) on the relative abundance of the 16S rRNA gene ASVs identified at the phylum level of *Bacteria*. ﻿

| **ANOVA test results** | | | | | | |
| --- | --- | --- | --- | --- | --- | --- |
| ***Acidobacteriota*** | **Df** | **Sum Sq** | **Mean Sq** | **F value** | **Pr(>F)** |  |
| **contamination** | **1** | **2.37E-03** | **2.37E-03** | **22.536** | **<0.001** | ******* |
| **compartment** | **1** | **4.75E-04** | **4.75E-04** | **4.526** | **0.035** | ***** |
| SFMIC | 7 | 5.41E-04 | 7.72E-05 | 0.736 | 0.642 |  |
| contamination:compartment | 1 | 9.50E-05 | 9.53E-05 | 0.908 | 0.342 |  |
| contamination:SFMIC | 7 | 8.08E-04 | 1.15E-04 | 1.1 | 0.366 |  |
| compartment:SFMIC | 7 | 6.24E-04 | 8.91E-05 | 0.849 | 0.549 |  |
| contamination:compartment:SFMIC | 7 | 3.19E-04 | 4.56E-05 | 0.435 | 0.879 |  |
| Residuals | 159 | 1.67E-02 | 1.05E-04 |  |  |  |
| ***Actinobacteriota*** | **Df** | **Sum Sq** | **Mean Sq** | **F value** | **Pr(>F)** |  |
| **contamination** | **1** | **1.64E-02** | **1.63E-02** | **15.628** | **<0.001** | ******* |
| **compartment** | **1** | **2.18E-02** | **2.18E-02** | **20.848** | **<0.001** | ******* |
| SFMIC | 7 | 9.13E-03 | 1.31E-03 | 1.247 | 0.280 |  |
| contamination:compartment | 1 | 6.10E-04 | 6.06E-04 | 0.58 | 0.448 |  |
| **contamination:SFMIC** | **7** | **1.29E-02** | **1.84E-03** | **1.757** | **0.100** | **.** |
| compartment:SFMIC | 7 | 5.79E-03 | 8.27E-04 | 0.79 | 0.596 |  |
| contamination:compartment:SFMIC | 7 | 3.96E-03 | 5.66E-04 | 0.541 | 0.803 |  |
| Residuals | 159 | 1.66E-01 | 1.05E-03 |  |  |  |
| ***Alphaproteobacteria*** | **Df** | **Sum Sq** | **Mean Sq** | **F value** | **Pr(>F)** |  |
| **contamination** | **1** | **1.30E-01** | **1.30E-01** | **58.382** | **<0.001** | ******* |
| **compartment** | **1** | **1.94E-02** | **1.94E-02** | **8.668** | **0.004** | ****** |
| SFMIC | 7 | 3.40E-03 | 4.90E-04 | 0.22 | 0.980 |  |
| **contamination:compartment** | **1** | **8.00E-03** | **8.00E-03** | **3.583** | **0.060** | **.** |
| contamination:SFMIC | 7 | 5.80E-03 | 8.30E-04 | 0.372 | 0.918 |  |
| compartment:SFMIC | 7 | 1.00E-02 | 1.42E-03 | 0.637 | 0.725 |  |
| contamination:compartment:SFMIC | 7 | 8.20E-03 | 1.17E-03 | 0.525 | 0.814 |  |
| Residuals | 159 | 3.55E-01 | 2.23E-03 |  |  |  |
| ***Bacteroidota*** | **Df** | **Sum Sq** | **Mean Sq** | **F value** | **Pr(>F)** |  |
| **contamination** | **1** | **1.00E-01** | **1.00E-01** | **72.515** | **<0.001** | ******* |
| compartment | 1 | 1.20E-03 | 1.20E-03 | 0.868 | 0.353 |  |
| SFMIC | 7 | 6.72E-03 | 9.60E-04 | 0.694 | 0.677 |  |
| contamination:compartment | 1 | 2.54E-03 | 2.54E-03 | 1.835 | 0.178 |  |
| **contamination:SFMIC** | **7** | **1.71E-02** | **2.44E-03** | **1.764** | **0.098** | **.** |
| compartment:SFMIC | 7 | 1.36E-02 | 1.95E-03 | 1.408 | 0.205 |  |
| contamination:compartment:SFMIC | 7 | 5.03E-03 | 7.20E-04 | 0.52 | 0.819 |  |
| Residuals | 159 | 2.20E-01 | 1.38E-03 |  |  |  |
| ***Bdellovibrionota*** | **Df** | **Sum Sq** | **Mean Sq** | **F value** | **Pr(>F)** |  |
| contamination | 1 | 1.00E-06 | 8.10E-07 | 0.035 | 0.853 |  |
| compartment | 1 | 2.10E-05 | 2.11E-05 | 0.905 | 0.343 |  |
| SFMIC | 7 | 2.33E-04 | 3.33E-05 | 1.428 | 0.197 |  |
| contamination:compartment | 1 | 1.00E-05 | 1.00E-05 | 0.429 | 0.514 |  |
| contamination:SFMIC | 7 | 1.36E-04 | 1.94E-05 | 0.832 | 0.563 |  |
| contamination:compartment:SFMIC | 7 | 5.40E-05 | 7.66E-06 | 0.328 | 0.94 |  |
| compartment:SFMIC | 7 | 8.10E-05 | 1.16E-05 | 0.498 | 0.835 |  |
| Residuals | 159 | 3.71E-03 | 2.33E-05 |  |  |  |
| ***Chloroflexota*** | **Df** | **Sum Sq** | **Mean Sq** | **F value** | **Pr(>F)** |  |
| **contamination** | **1** | **6.18E-02** | **6.18E-02** | **152.645** | **<0.001** | ******* |
| **compartment** | **1** | **5.32E-03** | **5.32E-03** | **13.132** | **<0.001** | ******* |
| SFMIC | 7 | 1.71E-03 | 2.40E-04 | 0.602 | 0.753 |  |
| contamination:compartment | 1 | 1.60E-04 | 1.60E-04 | 0.389 | 0.534 |  |
| contamination:SFMIC | 7 | 4.23E-03 | 6.00E-04 | 1.493 | 0.173 |  |
| compartment:SFMIC | 7 | 2.73E-03 | 3.90E-04 | 0.965 | 0.459 |  |
| contamination:compartment:SFMIC | 7 | 5.60E-04 | 8.00E-05 | 0.196 | 0.986 |  |
| Residuals | 159 |  |  |  |  |  |
| ***Firmicutes*** | **Df** | **Sum Sq** | **Mean Sq** | **F value** | **Pr(>F)** |  |
| contamination | 1 | 1.20E-04 | 1.20E-04 | 0.208 | 0.649 |  |
| **compartment** | **1** | **5.86E-03** | **5.86E-03** | **10.175** | **0.002** | ****** |
| SFMIC | 7 | 6.35E-03 | 9.07E-04 | 1.575 | 0.146 |  |
| contamination:compartment | 1 | 1.41E-03 | 1.41E-03 | 2.454 | 0.119 |  |
| contamination:SFMIC | 7 | 2.21E-03 | 3.16E-04 | 0.549 | 0.796 |  |
| compartment:SFMIC | 7 | 1.79E-03 | 2.56E-04 | 0.444 | 0.873 |  |
| contamination:compartment:SFMIC | 7 | 3.75E-03 | 5.36E-04 | 0.931 | 0.485 |  |
| Residuals | 159 | 9.16E-02 | 5.76E-04 |  |  |  |
| ***Gammaproteobacteria*** | **Df** | **Sum Sq** | **Mean Sq** | **F value** | **Pr(>F)** |  |
| **contamination** | **1** | **9.54E-02** | **9.54E-02** | **41.855** | **<0.001** | ******* |
| compartment | 1 | 0.00E+00 | 2.00E-05 | 0.007 | 0.934 |  |
| SFMIC | 7 | 1.22E-02 | 1.74E-03 | 0.762 | 0.620 |  |
| contamination:compartment | 1 | 2.00E-04 | 2.00E-04 | 0.089 | 0.766 |  |
| contamination:SFMIC | 7 | 1.49E-02 | 2.13E-03 | 0.933 | 0.483 |  |
| compartment:SFMIC | 7 | 8.60E-03 | 1.23E-03 | 0.538 | 0.805 |  |
| contamination:compartment:SFMIC | 7 | 3.70E-03 | 5.30E-04 | 0.234 | 0.976 |  |
| Residuals | 159 | 3.62E-01 | 2.28E-03 |  |  |  |
| ***Gemmatimonadota*** | **Df** | **Sum Sq** | **Mean Sq** | **F value** | **Pr(>F)** |  |
| **contamination** | **1** | **1.77E-03** | **1.77E-03** | **27.93** | **<0.001** | ******* |
| **compartment** | **1** | **1.06E-03** | **1.06E-03** | **16.681** | **<0.001** | ******* |
| SFMIC | 7 | 3.51E-04 | 5.02E-05 | 0.791 | 0.596 |  |
| **contamination:compartment** | **1** | **3.57E-04** | **3.57E-04** | **5.621** | **0.019** | ***** |
| contamination:SFMIC | 7 | 4.65E-04 | 6.64E-05 | 1.047 | 0.401 |  |
| compartment:SFMIC | 7 | 5.01E-04 | 7.16E-05 | 1.128 | 0.348 |  |
| contamination:compartment:SFMIC | 7 | 4.20E-04 | 5.99E-05 | 0.945 | 0.474 |  |
| Residuals | 159 | 1.01E-02 | 6.35E-05 |  |  |  |
| ***Myxococcota*** | **Df** | **Sum Sq** | **Mean Sq** | **F value** | **Pr(>F)** |  |
| contamination | 1 | 1.26E-04 | 1.26E-04 | 0.876 | 0.351 |  |
| compartment | 1 | 1.00E-06 | 1.23E-06 | 0.009 | 0.926 |  |
| SFMIC | 7 | 1.67E-03 | 2.39E-04 | 1.665 | 0.121 |  |
| contamination:compartment | 1 | 6.90E-05 | 6.87E-05 | 0.48 | 0.49 |  |
| contamination:SFMIC | 7 | 1.11E-03 | 1.59E-04 | 1.108 | 0.361 |  |
| compartment:SFMIC | 7 | 2.43E-04 | 3.48E-05 | 0.243 | 0.974 |  |
| contamination:compartment:SFMIC | 7 | 5.50E-04 | 7.86E-05 | 0.548 | 0.797 |  |
| Residuals | 159 | 2.28E-02 | 1.43E-04 |  |  |  |
| ***Planctomycetota*** | **Df** | **Sum Sq** | **Mean Sq** | **F value** | **Pr(>F)** |  |
| contamination | 1 | 2.00E-06 | 1.80E-06 | 0.027 | 0.869 |  |
| **compartment** | **1** | **7.24E-04** | **7.24E-04** | **10.69** | **0.001** | ****** |
| SFMIC | 7 | 6.13E-04 | 8.76E-05 | 1.294 | 0.257 |  |
| contamination:compartment | 1 | 1.00E-06 | 1.00E-06 | 0.015 | 0.904 |  |
| contamination:SFMIC | 7 | 4.95E-04 | 7.07E-05 | 1.044 | 0.403 |  |
| compartment:SFMIC | 7 | 6.23E-04 | 8.90E-05 | 1.314 | 0.247 |  |
| contamination:compartment:SFMIC | 7 | 4.12E-04 | 5.89E-05 | 0.87 | 0.532 |  |
| Residuals | 159 | 1.08E-02 | 6.77E-05 |  |  |  |
| ***Verrucomicrobiota*** | **Df** | **Sum Sq** | **Mean Sq** | **F value** | **Pr(>F)** |  |
| contamination | 1 | 3.26E-04 | 3.26E-04 | 1.966 | 0.163 |  |
| **compartment** | **1** | **3.78E-03** | **3.78E-03** | **22.764** | **<0.001** | ******* |
| SFMIC | 7 | 1.03E-03 | 1.47E-04 | 0.887 | 0.519 |  |
| contamination:compartment | 1 | 1.49E-04 | 1.49E-04 | 0.894 | 0.346 |  |
| contamination:SFMIC | 7 | 7.35E-04 | 1.05E-04 | 0.632 | 0.729 |  |
| compartment:SFMIC | 7 | 3.44E-04 | 4.90E-05 | 0.296 | 0.955 |  |
| **contamination:compartment:SFMIC** | **7** | **2.20E-03** | **3.14E-04** | **1.892** | **0.074** | **.** |
| Residuals | 159 | 2.64E-02 | 1.66E-04 |  |  |  |

^a^ Values in bold indicate significant or marginally significant effects. Df, degrees of freedom; F, variance ratio; Pr(>F), P value.

**S8 Table.** Summary of the three-way analysis of the variance (ANOVA) on the relative abundance of the PAH-RHDα Gram-negative gene ASVs identified at the genus level of *Bacteria*. ﻿

| **ANOVA test results** | | | | | | |
| --- | --- | --- | --- | --- | --- | --- |
| ***Aeromonas*** | **Df** | **Sum Sq** | **Mean Sq** | **F value** | **Pr(>F)** |  |
| **contamination** | **1** | **0.004** | **0.004** | **5.668** | **0.019** | ***** |
| compartment | 1 | 0.001 | 0.001 | 2.16 | 0.144 |  |
| SFMIC | 7 | 0.003 | 0 | 0.629 | 0.731 |  |
| contamination:compartment | 1 | 0 | 0 | 0.005 | 0.945 |  |
| contamination:SFMIC | 7 | 0.001 | 0 | 0.322 | 0.943 |  |
| compartment:SFMIC | 7 | 0.005 | 0.001 | 1.201 | 0.305 |  |
| contamination:compartment:SFMIC | 7 | 0.003 | 0 | 0.607 | 0.75 |  |
| Residuals | 151 | 0.096 | 0.001 |  |  |  |
| ***Bacillus*** | **Df** | **Sum Sq** | **Mean Sq** | **F value** | **Pr(>F)** |  |
| **contamination** | **1** | **0.007** | **0.007** | **4.984** | **0.027** | ***** |
| compartment | 1 | 0.003 | 0.003 | 2.391 | 0.124 |  |
| SFMIC | 7 | 0.01 | 0.001 | 1.058 | 0.394 |  |
| **contamination:compartment** | **1** | **0.004** | **0.004** | **3.019** | **0.084** | **.** |
| contamination:SFMIC | 7 | 0.01 | 0.001 | 1.076 | 0.381 |  |
| compartment:SFMIC | 7 | 0.006 | 0.001 | 0.642 | 0.721 |  |
| contamination:compartment:SFMIC | 7 | 0.003 | 0 | 0.36 | 0.924 |  |
| Residuals | 151 | 0.205 | 0.001 |  |  |  |
| ***Comamonas*** | **Df** | **Sum Sq** | **Mean Sq** | **F value** | **Pr(>F)** |  |
| **contamination** | **1** | **3.864** | **3.864** | **29.762** | **<0.001** | ******* |
| **compartment** | **1** | **0.535** | **0.535** | **4.122** | **0.044** | ***** |
| SFMIC | 7 | 0.353 | 0.05 | 0.389 | 0.908 |  |
| contamination:compartment | 1 | 0.154 | 0.154 | 1.19 | 0.277 |  |
| contamination:SFMIC | 7 | 1.013 | 0.145 | 1.114 | 0.357 |  |
| compartment:SFMIC | 7 | 0.66 | 0.094 | 0.726 | 0.65 |  |
| contamination:compartment:SFMIC | 7 | 0.232 | 0.033 | 0.256 | 0.97 |  |
| Residuals | 151 | 19.604 | 0.13 |  |  |  |
| ***Delftia*** | **Df** | **Sum Sq** | **Mean Sq** | **F value** | **Pr(>F)** |  |
| **contamination** | **1** | **9.226** | **9.226** | **93.241** | **<0.001** | ******* |
| **compartment** | **1** | **1.168** | **1.168** | **11.803** | **<0.001** | ******* |
| SFMIC | 7 | 0.185 | 0.026 | 0.268 | 0.966 |  |
| contamination:compartment | 1 | 0.269 | 0.269 | 2.718 | 0.101 |  |
| contamination:SFMIC | 7 | 0.807 | 0.115 | 1.166 | 0.326 |  |
| compartment:SFMIC | 7 | 0.403 | 0.058 | 0.582 | 0.77 |  |
| contamination:compartment:SFMIC | 7 | 0.096 | 0.014 | 0.138 | 0.995 |  |
| Residuals | 151 | 14.941 | 0.099 |  |  |  |
| ***Enterobacter*** | **Df** | **Sum Sq** | **Mean Sq** | **F value** | **Pr(>F)** |  |
| **contamination** | **1** | **0.005** | **0.005** | **5.119** | **0.025** | ***** |
| compartment | 1 | 0 | 0 | 0.199 | 0.656 |  |
| SFMIC | 7 | 0.003 | 0 | 0.423 | 0.887 |  |
| contamination:compartment | 1 | 0 | 0 | 0.397 | 0.53 |  |
| contamination:SFMIC | 7 | 0.004 | 0.001 | 0.544 | 0.8 |  |
| compartment:SFMIC | 7 | 0.008 | 0.001 | 1.047 | 0.401 |  |
| contamination:compartment:SFMIC | 7 | 0.005 | 0.001 | 0.729 | 0.647 |  |
| Residuals | 151 | 0.16 | 0.001 |  |  |  |
| ***Gammaproteobacteriota undef.*** | **Df** | **Sum Sq** | **Mean Sq** | **F value** | **Pr(>F)** |  |
| contamination | 1 | 0.016 | 0.016 | 1.705 | 0.194 |  |
| compartment | 1 | 0.012 | 0.012 | 1.24 | 0.267 |  |
| SFMIC | 7 | 0.037 | 0.005 | 0.564 | 0.784 |  |
| contamination:compartment | 1 | 0.008 | 0.008 | 0.863 | 0.354 |  |
| contamination:SFMIC | 7 | 0.055 | 0.008 | 0.831 | 0.563 |  |
| compartment:SFMIC | 7 | 0.064 | 0.009 | 0.97 | 0.456 |  |
| contamination:compartment:SFMIC | 7 | 0.064 | 0.009 | 0.965 | 0.459 |  |
| Residuals | 151 | 1.424 | 0.009 |  |  |  |
| ***Martelella*** | **Df** | **Sum Sq** | **Mean Sq** | **F value** | **Pr(>F)** |  |
| **contamination** | **1** | **0.008** | **0.008** | **5.67** | **0.019** | ***** |
| compartment | 1 | 0.001 | 0.001 | 0.533 | 0.467 |  |
| SFMIC | 7 | 0.006 | 0.001 | 0.594 | 0.76 |  |
| contamination:compartment | 1 | 0 | 0 | 0.095 | 0.758 |  |
| contamination:SFMIC | 7 | 0.004 | 0.001 | 0.449 | 0.87 |  |
| compartment:SFMIC | 7 | 0.012 | 0.002 | 1.26 | 0.274 |  |
| contamination:compartment:SFMIC | 7 | 0.003 | 0 | 0.342 | 0.933 |  |
| Residuals | 151 | 0.211 | 0.001 |  |  |  |
| **Other *Bacteria*** | **Df** | **Sum Sq** | **Mean Sq** | **F value** | **Pr(>F)** |  |
| **contamination** | **1** | **0.003** | **0.003** | **7.064** | **0.009** | ****** |
| compartment | 1 | 0.001 | 0.001 | 1.917 | 0.168 |  |
| SFMIC | 7 | 0.002 | 0 | 0.683 | 0.686 |  |
| contamination:compartment | 1 | 0 | 0 | 0.411 | 0.522 |  |
| contamination:SFMIC | 7 | 0.001 | 0 | 0.477 | 0.85 |  |
| compartment:SFMIC | 7 | 0.002 | 0 | 0.692 | 0.678 |  |
| contamination:compartment:SFMIC | 7 | 0.002 | 0 | 0.771 | 0.613 |  |
| Residuals | 151 | 0.061 | 0 |  |  |  |
| **Other *Proteobacteriota*** | **Df** | **Sum Sq** | **Mean Sq** | **F value** | **Pr(>F)** |  |
| **contamination** | **1** | **0.005** | **0.005** | **9.285** | **0.003** | ****** |
| **compartment** | **1** | **0.002** | **0.002** | **3.739** | **0.055** | **.** |
| SFMIC | 7 | 0.003 | 0 | 0.837 | 0.558 |  |
| contamination:compartment | 1 | 0.001 | 0.001 | 2.098 | 0.15 |  |
| contamination:SFMIC | 7 | 0.001 | 0 | 0.367 | 0.92 |  |
| compartment:SFMIC | 7 | 0.002 | 0 | 0.564 | 0.784 |  |
| contamination:compartment:SFMIC | 7 | 0.002 | 0 | 0.602 | 0.753 |  |
| Residuals | 151 | 0.084 | 0.001 |  |  |  |
| ***Pseudomonas*** | **Df** | **Sum Sq** | **Mean Sq** | **F value** | **Pr(>F)** |  |
| contamination | 1 | 0.007 | 0.007 | 1.655 | 0.2 |  |
| compartment | 1 | 0.002 | 0.002 | 0.391 | 0.532 |  |
| SFMIC | 7 | 0.026 | 0.004 | 0.851 | 0.547 |  |
| contamination:compartment | 1 | 0.002 | 0.002 | 0.424 | 0.516 |  |
| contamination:SFMIC | 7 | 0.027 | 0.004 | 0.883 | 0.521 |  |
| compartment:SFMIC | 7 | 0.032 | 0.005 | 1.027 | 0.415 |  |
| contamination:compartment:SFMIC | 7 | 0.032 | 0.005 | 1.052 | 0.397 |  |
| Residuals | 151 | 0.664 | 0.004 |  |  |  |
| ***Rahnella*** | **Df** | **Sum Sq** | **Mean Sq** | **F value** | **Pr(>F)** |  |
| contamination | 1 | 0.003 | 0.003 | 0.813 | 0.369 |  |
| compartment | 1 | 0.003 | 0.003 | 0.611 | 0.436 |  |
| SFMIC | 7 | 0.022 | 0.003 | 0.764 | 0.618 |  |
| contamination:compartment | 1 | 0.011 | 0.011 | 2.698 | 0.103 |  |
| contamination:SFMIC | 7 | 0.034 | 0.005 | 1.163 | 0.327 |  |
| compartment:SFMIC | 7 | 0.027 | 0.004 | 0.904 | 0.505 |  |
| contamination:compartment:SFMIC | 7 | 0.019 | 0.003 | 0.657 | 0.708 |  |
| Residuals | 151 | 0.633 | 0.004 |  |  |  |
| ***Ralstonia*** | **Df** | **Sum Sq** | **Mean Sq** | **F value** | **Pr(>F)** |  |
| contamination | 1 | 0.028 | 0.028 | 2.6 | 0.109 |  |
| compartment | 1 | 0 | 0 | 0.037 | 0.849 |  |
| SFMIC | 7 | 0.056 | 0.008 | 0.742 | 0.637 |  |
| contamination:compartment | 1 | 0 | 0 | 0.039 | 0.843 |  |
| contamination:SFMIC | 7 | 0.047 | 0.007 | 0.629 | 0.732 |  |
| compartment:SFMIC | 7 | 0.085 | 0.012 | 1.129 | 0.348 |  |
| contamination:compartment:SFMIC | 7 | 0.065 | 0.009 | 0.863 | 0.538 |  |
| Residuals | 151 | 1.616 | 0.011 |  |  |  |
| ***Shewanella*** | **Df** | **Sum Sq** | **Mean Sq** | **F value** | **Pr(>F)** |  |
| **contamination** | **1** | **0.005** | **0.005** | **6.755** | **0.01** | ***** |
| compartment | 1 | 0.001 | 0.001 | 0.873 | 0.352 |  |
| SFMIC | 7 | 0.003 | 0 | 0.588 | 0.765 |  |
| contamination:compartment | 1 | 0 | 0 | 0.019 | 0.89 |  |
| contamination:SFMIC | 7 | 0.001 | 0 | 0.201 | 0.985 |  |
| compartment:SFMIC | 7 | 0.007 | 0.001 | 1.359 | 0.227 |  |
| contamination:compartment:SFMIC | 7 | 0.003 | 0 | 0.594 | 0.76 |  |
| Residuals | 151 | 0.11 | 0.001 |  |  |  |

^a^ Values in bold indicate significant or marginally significant effects. Df, degrees of freedom; F, variance ratio; Pr(>F), P value.

**S9 Table.** Summary of the three-way analysis of the variance (ANOVA) on the relativeabundance of the PAH-RHDα Gram-positive gene ASVs identified at the genus level of *Bacteria*. ﻿

| **ANOVA test results** | | | | | | |
| --- | --- | --- | --- | --- | --- | --- |
| ***Other Bacteria*** | **Df** | **Sum Sq** | **Mean Sq** | **F value** | **Pr(>F)** |  |
| **contamination** | **1** | **0.243** | **0.243** | **61.471** | **<0.001** | ******* |
| compartment | 1 | 0.002 | 0.002 | 0.442 | 0.507 |  |
| **SFMIC** | **7** | **0.049** | **0.007** | **1.777** | **0.096** | **.** |
| contamination:compartment | 1 | 0.001 | 0.001 | 0.197 | 0.658 |  |
| contamination:SFMIC | 7 | 0.045 | 0.006 | 1.631 | 0.131 |  |
| compartment:SFMIC | 7 | 0.041 | 0.006 | 1.492 | 0.174 |  |
| contamination:compartment:SFMIC | 7 | 0.036 | 0.005 | 1.285 | 0.261 |  |
| Residuals | 153 | 0.605 | 0.004 |  |  |  |
| ***Other Actinobacteriota*** | **Df** | **Sum Sq** | **Mean Sq** | **F value** | **Pr(>F)** |  |
| **contamination** | **1** | **0.129** | **0.129** | **52.503** | **<0.001** | ******* |
| compartment | 1 | 0.003 | 0.003 | 1.063 | 0.304 |  |
| SFMIC | 7 | 0.006 | 0.001 | 0.375 | 0.916 |  |
| contamination:compartment | 1 | 0.005 | 0.005 | 2.080 | 0.151 |  |
| contamination:SFMIC | 7 | 0.017 | 0.002 | 0.965 | 0.459 |  |
| **compartment:SFMIC** | **7** | **0.035** | **0.005** | **2.007** | **0.058** | **.** |
| contamination:compartment:SFMIC | 7 | 0.025 | 0.004 | 1.450 | 0.189 |  |
| Residuals | 153 | 0.376 | 0.002 |  |  |  |
| ***Actinobacteria undef.*** | **Df** | **Sum Sq** | **Mean Sq** | **F value** | **Pr(>F)** |  |
| **contamination** | **1** | **0.004** | **0.004** | **17.667** | **<0.001** | ******* |
| **compartment** | **1** | **0.012** | **0.012** | **56.827** | **<0.001** | ******* |
| SFMIC | 7 | 0.000 | 0.000 | 0.188 | 0.988 |  |
| contamination:compartment | 1 | 0.000 | 0.000 | 0.197 | 0.658 |  |
| contamination:SFMIC | 7 | 0.001 | 0.000 | 0.981 | 0.447 |  |
| compartment:SFMIC | 7 | 0.002 | 0.000 | 1.161 | 0.328 |  |
| **contamination:compartment:SFMIC** | **7** | **0.004** | **0.001** | **2.437** | **0.022** | ***** |
| Residuals | 153 | 0.032 | 0.000 |  |  |  |
| ***Mycobacterium*** | **Df** | **Sum Sq** | **Mean Sq** | **F value** | **Pr(>F)** |  |
| **contamination** | **1** | **0.026** | **0.026** | **44.519** | **<0.001** | ******* |
| compartment | 1 | 0.001 | 0.001 | 2.021 | 0.157 |  |
| SFMIC | 7 | 0.002 | 0.000 | 0.506 | 0.829 |  |
| contamination:compartment | 1 | 0.002 | 0.002 | 2.713 | 0.102 |  |
| contamination:SFMIC | 7 | 0.002 | 0.000 | 0.607 | 0.749 |  |
| compartment:SFMIC | 7 | 0.001 | 0.000 | 0.263 | 0.967 |  |
| contamination:compartment:SFMIC | 7 | 0.001 | 0.000 | 0.138 | 0.995 |  |
| Residuals | 153 | 0.089 | 0.001 |  |  |  |
| ***Microbacterium*** | **Df** | **Sum Sq** | **Mean Sq** | **F value** | **Pr(>F)** |  |
| contamination | 1 | 0.008 | 0.008 | 1.981 | 0.161 |  |
| compartment | 1 | 0.001 | 0.001 | 0.318 | 0.574 |  |
| SFMIC | 7 | 0.033 | 0.005 | 1.105 | 0.363 |  |
| contamination:compartment | 1 | 0.008 | 0.008 | 1.810 | 0.181 |  |
| contamination:SFMIC | 7 | 0.024 | 0.003 | 0.789 | 0.598 |  |
| compartment:SFMIC | 7 | 0.026 | 0.004 | 0.863 | 0.537 |  |
| contamination:compartment:SFMIC | 7 | 0.031 | 0.004 | 1.048 | 0.400 |  |
| Residuals | 153 | 0.652 | 0.004 |  |  |  |
| ***Other Proteobacteriota*** | **Df** | **Sum Sq** | **Mean Sq** | **F value** | **Pr(>F)** |  |
| **contamination** | **1** | **0.007** | **0.007** | **41.720** | **<0.001** | ******* |
| **compartment** | **1** | **0.003** | **0.003** | **17.525** | **<0.001** | ******* |
| SFMIC | 7 | 0.001 | 0.000 | 0.827 | 0.566 |  |
| **contamination:compartment** | **1** | **0.002** | **0.002** | **13.868** | **<0.001** | ******* |
| contamination:SFMIC | 7 | 0.001 | 0.000 | 0.664 | 0.702 |  |
| compartment:SFMIC | 7 | 0.000 | 0.000 | 0.381 | 0.912 |  |
| contamination:compartment:SFMIC | 7 | 0.000 | 0.000 | 0.400 | 0.901 |  |
| Residuals | 153 | 0.025 | 0.000 |  |  |  |
| ***Other Micrococcales*** | **Df** | **Sum Sq** | **Mean Sq** | **F value** | **Pr(>F)** |  |
| **contamination** | **1** | **0.001** | **0.001** | **13.452** | **<0.001** | ******* |
| **compartment** | **1** | **0.000** | **0.000** | **3.446** | **0.065** | **.** |
| SFMIC | 7 | 0.000 | 0.000 | 0.348 | 0.930 |  |
| contamination:compartment | 1 | 0.000 | 0.000 | 0.294 | 0.588 |  |
| contamination:SFMIC | 7 | 0.000 | 0.000 | 0.321 | 0.944 |  |
| compartment:SFMIC | 7 | 0.000 | 0.000 | 0.467 | 0.857 |  |
| contamination:compartment:SFMIC | 7 | 0.000 | 0.000 | 0.452 | 0.868 |  |
| Residuals | 153 | 0.007 | 0.000 | 9.000 |  |  |
| ***Others*** | **Df** | **Sum Sq** | **Mean Sq** | **F value** | **Pr(>F)** |  |
| **contamination** | 1 | 0.005 | 0.005 | 17.183 | **<0.001** | *** |
| compartment | 1 | 0.002 | 0.002 | 7.899 | 0.006 | ** |
| SFMIC | 7 | 0.002 | 0.000 | 0.767 | 0.616 |  |
| **contamination:compartment** | **1** | **0.002** | **0.002** | **7.101** | **0.009** | ****** |
| contamination:SFMIC | 7 | 0.002 | 0.000 | 0.871 | 0.531 |  |
| compartment:SFMIC | 7 | 0.001 | 0.000 | 0.462 | 0.860 |  |
| contamination:compartment:SFMIC | 7 | 0.001 | 0.000 | 0.615 | 0.743 |  |
| Residuals | 153 | 0.048 | 0.000 |  |  |  |

^a^ Values in bold indicate significant or marginally significant effects. Df, degrees of freedom; F, variance ratio; Pr(>F), P value.

**S10 Table.** Summary of the three-way analysis of the variance (ANOVA) on the absolute abundance of PAH-RHD genes (on copy numbers per gram of soil) ^†^.

| ***Gram negative genes*** | ***Df*** | ***Sum Sq*** | ***Mean Sq*** | ***F value*** | ***Pr(>F)*** | |
| --- | --- | --- | --- | --- | --- | --- |
| SFMIC | 7 | 1.29E+18 | 1.84E+17 | 1.486 | 0.1757 |  |
| **contamination** | **1** | **2.86E+18** | **2.86E+18** | **23.124** | **<0.001** | ******* |
| **compartment** | **2** | **3.88E+18** | **1.94E+18** | **15.702** | **<0.001** | ******* |
| SFMIC:contamination | 7 | 6.73E+17 | 9.61E+16 | 0.778 | 0.6064 |  |
| SFMIC:compartment | 7 | 8.83E+17 | 1.26E+17 | 1.022 | 0.4181 |  |
| **contamination:compartment** | **1** | **3.91E+17** | **3.91E+17** | **3.166** | **0.0771** | **.** |
| SFMIC:contamination:compartment | 7 | 1.28E+18 | 1.82E+17 | 1.475 | 0.1799 |  |
| Residuals | 157 | 1.94E+19 | 1.24E+17 |  |  |  |
| ***Gram positive genes*** | ***Df*** | ***Sum Sq*** | ***Mean Sq*** | ***F value*** | ***Pr(>F)*** | |
| SFMIC | 7 | 2.43E+14 | 3.47E+13 | 0.423 | 0.887 |  |
| **contamination** | **1** | **1.30E+15** | **1.30E+15** | **15.758** | **<0.001** | ******* |
| **compartment** | **1** | **1.14E+15** | **5.70E+14** | **6.937** | **0.001** | ****** |
| SFMIC:contamination | 7 | 9.81E+14 | 1.40E+14 | 1.705 | 0.111 |  |
| SFMIC:compartment | 7 | 2.96E+14 | 4.23E+13 | 0.514 | 0.823 |  |
| **contamination:compartment** | **1** | **2.47E+14** | **2.47E+14** | **3.000** | **0.085** | **.** |
| SFMIC:contamination:compartment | 7 | 9.88E+14 | 1.41E+14 | 1.718 | 0.108 |  |
| Residuals | 159 | 1.29E+16 | 8.22E+13 |  |  |  |

^†^ Values in bold indicate significant or marginally significant effects. The number of asterisks denote the strength of the difference: * for p values < 0.05; *** for p values <0.001 and a dot instead of an asterisk to denote nearly significant difference. Df, degrees of freedom; F, variance ratio; Pr(>F), P value.

**S1 Fig**
