# Supplementary material for: Soil fauna-microbial interactions shifts fungal and bacterial communities under a contamination disturbance
Source: PLoS One. 2023 Oct 25;18(10):e0292227. doi: 10.1371/journal.pone.0292227 (PMC10599570; doi:10.1371/journal.pone.0292227)
Supplement: S8 Table — (DOCX) [file pone.0292227.s008.docx]

**Table S8.** Summary of the three-way analysis of the variance (ANOVA) on the relative abundance of the PAH-RHDα Gram-negative gene ASVs identified at the genus level of *Bacteria*. ﻿

| **ANOVA test results** | | | | | | |
| --- | --- | --- | --- | --- | --- | --- |
| ***Aeromonas*** | **Df** | **Sum Sq** | **Mean Sq** | **F value** | **Pr(>F)** |  |
| **contamination** | **1** | **0.004** | **0.004** | **5.668** | **0.019** | ***** |
| compartment | 1 | 0.001 | 0.001 | 2.16 | 0.144 |  |
| SFMIC | 7 | 0.003 | 0 | 0.629 | 0.731 |  |
| contamination:compartment | 1 | 0 | 0 | 0.005 | 0.945 |  |
| contamination:SFMIC | 7 | 0.001 | 0 | 0.322 | 0.943 |  |
| compartment:SFMIC | 7 | 0.005 | 0.001 | 1.201 | 0.305 |  |
| contamination:compartment:SFMIC | 7 | 0.003 | 0 | 0.607 | 0.75 |  |
| Residuals | 151 | 0.096 | 0.001 |  |  |  |
| ***Bacillus*** | **Df** | **Sum Sq** | **Mean Sq** | **F value** | **Pr(>F)** |  |
| **contamination** | **1** | **0.007** | **0.007** | **4.984** | **0.027** | ***** |
| compartment | 1 | 0.003 | 0.003 | 2.391 | 0.124 |  |
| SFMIC | 7 | 0.01 | 0.001 | 1.058 | 0.394 |  |
| **contamination:compartment** | **1** | **0.004** | **0.004** | **3.019** | **0.084** | **.** |
| contamination:SFMIC | 7 | 0.01 | 0.001 | 1.076 | 0.381 |  |
| compartment:SFMIC | 7 | 0.006 | 0.001 | 0.642 | 0.721 |  |
| contamination:compartment:SFMIC | 7 | 0.003 | 0 | 0.36 | 0.924 |  |
| Residuals | 151 | 0.205 | 0.001 |  |  |  |
| ***Comamonas*** | **Df** | **Sum Sq** | **Mean Sq** | **F value** | **Pr(>F)** |  |
| **contamination** | **1** | **3.864** | **3.864** | **29.762** | **<0.001** | ******* |
| **compartment** | **1** | **0.535** | **0.535** | **4.122** | **0.044** | ***** |
| SFMIC | 7 | 0.353 | 0.05 | 0.389 | 0.908 |  |
| contamination:compartment | 1 | 0.154 | 0.154 | 1.19 | 0.277 |  |
| contamination:SFMIC | 7 | 1.013 | 0.145 | 1.114 | 0.357 |  |
| compartment:SFMIC | 7 | 0.66 | 0.094 | 0.726 | 0.65 |  |
| contamination:compartment:SFMIC | 7 | 0.232 | 0.033 | 0.256 | 0.97 |  |
| Residuals | 151 | 19.604 | 0.13 |  |  |  |
| ***Delftia*** | **Df** | **Sum Sq** | **Mean Sq** | **F value** | **Pr(>F)** |  |
| **contamination** | **1** | **9.226** | **9.226** | **93.241** | **<0.001** | ******* |
| **compartment** | **1** | **1.168** | **1.168** | **11.803** | **<0.001** | ******* |
| SFMIC | 7 | 0.185 | 0.026 | 0.268 | 0.966 |  |
| contamination:compartment | 1 | 0.269 | 0.269 | 2.718 | 0.101 |  |
| contamination:SFMIC | 7 | 0.807 | 0.115 | 1.166 | 0.326 |  |
| compartment:SFMIC | 7 | 0.403 | 0.058 | 0.582 | 0.77 |  |
| contamination:compartment:SFMIC | 7 | 0.096 | 0.014 | 0.138 | 0.995 |  |
| Residuals | 151 | 14.941 | 0.099 |  |  |  |
| ***Enterobacter*** | **Df** | **Sum Sq** | **Mean Sq** | **F value** | **Pr(>F)** |  |
| **contamination** | **1** | **0.005** | **0.005** | **5.119** | **0.025** | ***** |
| compartment | 1 | 0 | 0 | 0.199 | 0.656 |  |
| SFMIC | 7 | 0.003 | 0 | 0.423 | 0.887 |  |
| contamination:compartment | 1 | 0 | 0 | 0.397 | 0.53 |  |
| contamination:SFMIC | 7 | 0.004 | 0.001 | 0.544 | 0.8 |  |
| compartment:SFMIC | 7 | 0.008 | 0.001 | 1.047 | 0.401 |  |
| contamination:compartment:SFMIC | 7 | 0.005 | 0.001 | 0.729 | 0.647 |  |
| Residuals | 151 | 0.16 | 0.001 |  |  |  |
| ***Gammaproteobacteriota undef.*** | **Df** | **Sum Sq** | **Mean Sq** | **F value** | **Pr(>F)** |  |
| contamination | 1 | 0.016 | 0.016 | 1.705 | 0.194 |  |
| compartment | 1 | 0.012 | 0.012 | 1.24 | 0.267 |  |
| SFMIC | 7 | 0.037 | 0.005 | 0.564 | 0.784 |  |
| contamination:compartment | 1 | 0.008 | 0.008 | 0.863 | 0.354 |  |
| contamination:SFMIC | 7 | 0.055 | 0.008 | 0.831 | 0.563 |  |
| compartment:SFMIC | 7 | 0.064 | 0.009 | 0.97 | 0.456 |  |
| contamination:compartment:SFMIC | 7 | 0.064 | 0.009 | 0.965 | 0.459 |  |
| Residuals | 151 | 1.424 | 0.009 |  |  |  |
| ***Martelella*** | **Df** | **Sum Sq** | **Mean Sq** | **F value** | **Pr(>F)** |  |
| **contamination** | **1** | **0.008** | **0.008** | **5.67** | **0.019** | ***** |
| compartment | 1 | 0.001 | 0.001 | 0.533 | 0.467 |  |
| SFMIC | 7 | 0.006 | 0.001 | 0.594 | 0.76 |  |
| contamination:compartment | 1 | 0 | 0 | 0.095 | 0.758 |  |
| contamination:SFMIC | 7 | 0.004 | 0.001 | 0.449 | 0.87 |  |
| compartment:SFMIC | 7 | 0.012 | 0.002 | 1.26 | 0.274 |  |
| contamination:compartment:SFMIC | 7 | 0.003 | 0 | 0.342 | 0.933 |  |
| Residuals | 151 | 0.211 | 0.001 |  |  |  |
| **Other *Bacteria*** | **Df** | **Sum Sq** | **Mean Sq** | **F value** | **Pr(>F)** |  |
| **contamination** | **1** | **0.003** | **0.003** | **7.064** | **0.009** | ****** |
| compartment | 1 | 0.001 | 0.001 | 1.917 | 0.168 |  |
| SFMIC | 7 | 0.002 | 0 | 0.683 | 0.686 |  |
| contamination:compartment | 1 | 0 | 0 | 0.411 | 0.522 |  |
| contamination:SFMIC | 7 | 0.001 | 0 | 0.477 | 0.85 |  |
| compartment:SFMIC | 7 | 0.002 | 0 | 0.692 | 0.678 |  |
| contamination:compartment:SFMIC | 7 | 0.002 | 0 | 0.771 | 0.613 |  |
| Residuals | 151 | 0.061 | 0 |  |  |  |
| **Other *Proteobacteriota*** | **Df** | **Sum Sq** | **Mean Sq** | **F value** | **Pr(>F)** |  |
| **contamination** | **1** | **0.005** | **0.005** | **9.285** | **0.003** | ****** |
| **compartment** | **1** | **0.002** | **0.002** | **3.739** | **0.055** | **.** |
| SFMIC | 7 | 0.003 | 0 | 0.837 | 0.558 |  |
| contamination:compartment | 1 | 0.001 | 0.001 | 2.098 | 0.15 |  |
| contamination:SFMIC | 7 | 0.001 | 0 | 0.367 | 0.92 |  |
| compartment:SFMIC | 7 | 0.002 | 0 | 0.564 | 0.784 |  |
| contamination:compartment:SFMIC | 7 | 0.002 | 0 | 0.602 | 0.753 |  |
| Residuals | 151 | 0.084 | 0.001 |  |  |  |
| ***Pseudomonas*** | **Df** | **Sum Sq** | **Mean Sq** | **F value** | **Pr(>F)** |  |
| contamination | 1 | 0.007 | 0.007 | 1.655 | 0.2 |  |
| compartment | 1 | 0.002 | 0.002 | 0.391 | 0.532 |  |
| SFMIC | 7 | 0.026 | 0.004 | 0.851 | 0.547 |  |
| contamination:compartment | 1 | 0.002 | 0.002 | 0.424 | 0.516 |  |
| contamination:SFMIC | 7 | 0.027 | 0.004 | 0.883 | 0.521 |  |
| compartment:SFMIC | 7 | 0.032 | 0.005 | 1.027 | 0.415 |  |
| contamination:compartment:SFMIC | 7 | 0.032 | 0.005 | 1.052 | 0.397 |  |
| Residuals | 151 | 0.664 | 0.004 |  |  |  |
| ***Rahnella*** | **Df** | **Sum Sq** | **Mean Sq** | **F value** | **Pr(>F)** |  |
| contamination | 1 | 0.003 | 0.003 | 0.813 | 0.369 |  |
| compartment | 1 | 0.003 | 0.003 | 0.611 | 0.436 |  |
| SFMIC | 7 | 0.022 | 0.003 | 0.764 | 0.618 |  |
| contamination:compartment | 1 | 0.011 | 0.011 | 2.698 | 0.103 |  |
| contamination:SFMIC | 7 | 0.034 | 0.005 | 1.163 | 0.327 |  |
| compartment:SFMIC | 7 | 0.027 | 0.004 | 0.904 | 0.505 |  |
| contamination:compartment:SFMIC | 7 | 0.019 | 0.003 | 0.657 | 0.708 |  |
| Residuals | 151 | 0.633 | 0.004 |  |  |  |
| ***Ralstonia*** | **Df** | **Sum Sq** | **Mean Sq** | **F value** | **Pr(>F)** |  |
| contamination | 1 | 0.028 | 0.028 | 2.6 | 0.109 |  |
| compartment | 1 | 0 | 0 | 0.037 | 0.849 |  |
| SFMIC | 7 | 0.056 | 0.008 | 0.742 | 0.637 |  |
| contamination:compartment | 1 | 0 | 0 | 0.039 | 0.843 |  |
| contamination:SFMIC | 7 | 0.047 | 0.007 | 0.629 | 0.732 |  |
| compartment:SFMIC | 7 | 0.085 | 0.012 | 1.129 | 0.348 |  |
| contamination:compartment:SFMIC | 7 | 0.065 | 0.009 | 0.863 | 0.538 |  |
| Residuals | 151 | 1.616 | 0.011 |  |  |  |
| ***Shewanella*** | **Df** | **Sum Sq** | **Mean Sq** | **F value** | **Pr(>F)** |  |
| **contamination** | **1** | **0.005** | **0.005** | **6.755** | **0.01** | ***** |
| compartment | 1 | 0.001 | 0.001 | 0.873 | 0.352 |  |
| SFMIC | 7 | 0.003 | 0 | 0.588 | 0.765 |  |
| contamination:compartment | 1 | 0 | 0 | 0.019 | 0.89 |  |
| contamination:SFMIC | 7 | 0.001 | 0 | 0.201 | 0.985 |  |
| compartment:SFMIC | 7 | 0.007 | 0.001 | 1.359 | 0.227 |  |
| contamination:compartment:SFMIC | 7 | 0.003 | 0 | 0.594 | 0.76 |  |
| Residuals | 151 | 0.11 | 0.001 |  |  |  |

^a^ Values in bold indicate significant or marginally significant effects. Df, degrees of freedom; F, variance ratio; Pr(>F), P value.
