# Supplementary material for: Soil fauna-microbial interactions shifts fungal and bacterial communities under a contamination disturbance
Source: PLoS One. 2023 Oct 25;18(10):e0292227. doi: 10.1371/journal.pone.0292227 (PMC10599570; doi:10.1371/journal.pone.0292227)
Supplement: S9 Table — (DOCX) [file pone.0292227.s009.docx]

**Table S9.** Summary of the three-way analysis of the variance (ANOVA) on the relativeabundance of the PAH-RHDα Gram-positive gene ASVs identified at the genus level of *Bacteria*. ﻿

| **ANOVA test results** | | | | | | |
| --- | --- | --- | --- | --- | --- | --- |
| ***Other Bacteria*** | **Df** | **Sum Sq** | **Mean Sq** | **F value** | **Pr(>F)** |  |
| **contamination** | **1** | **0.243** | **0.243** | **61.471** | **<0.001** | ******* |
| compartment | 1 | 0.002 | 0.002 | 0.442 | 0.507 |  |
| **SFMIC** | **7** | **0.049** | **0.007** | **1.777** | **0.096** | **.** |
| contamination:compartment | 1 | 0.001 | 0.001 | 0.197 | 0.658 |  |
| contamination:SFMIC | 7 | 0.045 | 0.006 | 1.631 | 0.131 |  |
| compartment:SFMIC | 7 | 0.041 | 0.006 | 1.492 | 0.174 |  |
| contamination:compartment:SFMIC | 7 | 0.036 | 0.005 | 1.285 | 0.261 |  |
| Residuals | 153 | 0.605 | 0.004 |  |  |  |
| ***Other Actinobacteriota*** | **Df** | **Sum Sq** | **Mean Sq** | **F value** | **Pr(>F)** |  |
| **contamination** | **1** | **0.129** | **0.129** | **52.503** | **<0.001** | ******* |
| compartment | 1 | 0.003 | 0.003 | 1.063 | 0.304 |  |
| SFMIC | 7 | 0.006 | 0.001 | 0.375 | 0.916 |  |
| contamination:compartment | 1 | 0.005 | 0.005 | 2.080 | 0.151 |  |
| contamination:SFMIC | 7 | 0.017 | 0.002 | 0.965 | 0.459 |  |
| **compartment:SFMIC** | **7** | **0.035** | **0.005** | **2.007** | **0.058** | **.** |
| contamination:compartment:SFMIC | 7 | 0.025 | 0.004 | 1.450 | 0.189 |  |
| Residuals | 153 | 0.376 | 0.002 |  |  |  |
| ***Actinobacteria undef.*** | **Df** | **Sum Sq** | **Mean Sq** | **F value** | **Pr(>F)** |  |
| **contamination** | **1** | **0.004** | **0.004** | **17.667** | **<0.001** | ******* |
| **compartment** | **1** | **0.012** | **0.012** | **56.827** | **<0.001** | ******* |
| SFMIC | 7 | 0.000 | 0.000 | 0.188 | 0.988 |  |
| contamination:compartment | 1 | 0.000 | 0.000 | 0.197 | 0.658 |  |
| contamination:SFMIC | 7 | 0.001 | 0.000 | 0.981 | 0.447 |  |
| compartment:SFMIC | 7 | 0.002 | 0.000 | 1.161 | 0.328 |  |
| **contamination:compartment:SFMIC** | **7** | **0.004** | **0.001** | **2.437** | **0.022** | ***** |
| Residuals | 153 | 0.032 | 0.000 |  |  |  |
| ***Mycobacterium*** | **Df** | **Sum Sq** | **Mean Sq** | **F value** | **Pr(>F)** |  |
| **contamination** | **1** | **0.026** | **0.026** | **44.519** | **<0.001** | ******* |
| compartment | 1 | 0.001 | 0.001 | 2.021 | 0.157 |  |
| SFMIC | 7 | 0.002 | 0.000 | 0.506 | 0.829 |  |
| contamination:compartment | 1 | 0.002 | 0.002 | 2.713 | 0.102 |  |
| contamination:SFMIC | 7 | 0.002 | 0.000 | 0.607 | 0.749 |  |
| compartment:SFMIC | 7 | 0.001 | 0.000 | 0.263 | 0.967 |  |
| contamination:compartment:SFMIC | 7 | 0.001 | 0.000 | 0.138 | 0.995 |  |
| Residuals | 153 | 0.089 | 0.001 |  |  |  |
| ***Microbacterium*** | **Df** | **Sum Sq** | **Mean Sq** | **F value** | **Pr(>F)** |  |
| contamination | 1 | 0.008 | 0.008 | 1.981 | 0.161 |  |
| compartment | 1 | 0.001 | 0.001 | 0.318 | 0.574 |  |
| SFMIC | 7 | 0.033 | 0.005 | 1.105 | 0.363 |  |
| contamination:compartment | 1 | 0.008 | 0.008 | 1.810 | 0.181 |  |
| contamination:SFMIC | 7 | 0.024 | 0.003 | 0.789 | 0.598 |  |
| compartment:SFMIC | 7 | 0.026 | 0.004 | 0.863 | 0.537 |  |
| contamination:compartment:SFMIC | 7 | 0.031 | 0.004 | 1.048 | 0.400 |  |
| Residuals | 153 | 0.652 | 0.004 |  |  |  |
| ***Other Proteobacteriota*** | **Df** | **Sum Sq** | **Mean Sq** | **F value** | **Pr(>F)** |  |
| **contamination** | **1** | **0.007** | **0.007** | **41.720** | **<0.001** | ******* |
| **compartment** | **1** | **0.003** | **0.003** | **17.525** | **<0.001** | ******* |
| SFMIC | 7 | 0.001 | 0.000 | 0.827 | 0.566 |  |
| **contamination:compartment** | **1** | **0.002** | **0.002** | **13.868** | **<0.001** | ******* |
| contamination:SFMIC | 7 | 0.001 | 0.000 | 0.664 | 0.702 |  |
| compartment:SFMIC | 7 | 0.000 | 0.000 | 0.381 | 0.912 |  |
| contamination:compartment:SFMIC | 7 | 0.000 | 0.000 | 0.400 | 0.901 |  |
| Residuals | 153 | 0.025 | 0.000 |  |  |  |
| ***Other Micrococcales*** | **Df** | **Sum Sq** | **Mean Sq** | **F value** | **Pr(>F)** |  |
| **contamination** | **1** | **0.001** | **0.001** | **13.452** | **<0.001** | ******* |
| **compartment** | **1** | **0.000** | **0.000** | **3.446** | **0.065** | **.** |
| SFMIC | 7 | 0.000 | 0.000 | 0.348 | 0.930 |  |
| contamination:compartment | 1 | 0.000 | 0.000 | 0.294 | 0.588 |  |
| contamination:SFMIC | 7 | 0.000 | 0.000 | 0.321 | 0.944 |  |
| compartment:SFMIC | 7 | 0.000 | 0.000 | 0.467 | 0.857 |  |
| contamination:compartment:SFMIC | 7 | 0.000 | 0.000 | 0.452 | 0.868 |  |
| Residuals | 153 | 0.007 | 0.000 | 9.000 |  |  |
| ***Others*** | **Df** | **Sum Sq** | **Mean Sq** | **F value** | **Pr(>F)** |  |
| **contamination** | 1 | 0.005 | 0.005 | 17.183 | **<0.001** | *** |
| compartment | 1 | 0.002 | 0.002 | 7.899 | 0.006 | ** |
| SFMIC | 7 | 0.002 | 0.000 | 0.767 | 0.616 |  |
| **contamination:compartment** | **1** | **0.002** | **0.002** | **7.101** | **0.009** | ****** |
| contamination:SFMIC | 7 | 0.002 | 0.000 | 0.871 | 0.531 |  |
| compartment:SFMIC | 7 | 0.001 | 0.000 | 0.462 | 0.860 |  |
| contamination:compartment:SFMIC | 7 | 0.001 | 0.000 | 0.615 | 0.743 |  |
| Residuals | 153 | 0.048 | 0.000 |  |  |  |

^a^ Values in bold indicate significant or marginally significant effects. Df, degrees of freedom; F, variance ratio; Pr(>F), P value.
