# Supplementary material for: Soil fauna-microbial interactions shifts fungal and bacterial communities under a contamination disturbance
Source: PLoS One. 2023 Oct 25;18(10):e0292227. doi: 10.1371/journal.pone.0292227 (PMC10599570; doi:10.1371/journal.pone.0292227)
Supplement: S10 Table — (DOCX) [file pone.0292227.s010.docx]

**Table S10.** Summary of the three-way analysis of the variance (ANOVA) on the absolute abundance of PAH-RHD genes (on copy numbers per gram of soil) ^†^.

| ***Gram negative genes*** | ***Df*** | ***Sum Sq*** | ***Mean Sq*** | ***F value*** | ***Pr(>F)*** | |
| --- | --- | --- | --- | --- | --- | --- |
| SFMIC | 7 | 1.29E+18 | 1.84E+17 | 1.486 | 0.1757 |  |
| **contamination** | **1** | **2.86E+18** | **2.86E+18** | **23.124** | **<0.001** | ******* |
| **compartment** | **2** | **3.88E+18** | **1.94E+18** | **15.702** | **<0.001** | ******* |
| SFMIC:contamination | 7 | 6.73E+17 | 9.61E+16 | 0.778 | 0.6064 |  |
| SFMIC:compartment | 7 | 8.83E+17 | 1.26E+17 | 1.022 | 0.4181 |  |
| **contamination:compartment** | **1** | **3.91E+17** | **3.91E+17** | **3.166** | **0.0771** | **.** |
| SFMIC:contamination:compartment | 7 | 1.28E+18 | 1.82E+17 | 1.475 | 0.1799 |  |
| Residuals | 157 | 1.94E+19 | 1.24E+17 |  |  |  |
| ***Gram positive genes*** | ***Df*** | ***Sum Sq*** | ***Mean Sq*** | ***F value*** | ***Pr(>F)*** | |
| SFMIC | 7 | 2.43E+14 | 3.47E+13 | 0.423 | 0.887 |  |
| **contamination** | **1** | **1.30E+15** | **1.30E+15** | **15.758** | **<0.001** | ******* |
| **compartment** | **1** | **1.14E+15** | **5.70E+14** | **6.937** | **0.001** | ****** |
| SFMIC:contamination | 7 | 9.81E+14 | 1.40E+14 | 1.705 | 0.111 |  |
| SFMIC:compartment | 7 | 2.96E+14 | 4.23E+13 | 0.514 | 0.823 |  |
| **contamination:compartment** | **1** | **2.47E+14** | **2.47E+14** | **3.000** | **0.085** | **.** |
| SFMIC:contamination:compartment | 7 | 9.88E+14 | 1.41E+14 | 1.718 | 0.108 |  |
| Residuals | 159 | 1.29E+16 | 8.22E+13 |  |  |  |

^†^ Values in bold indicate significant or marginally significant effects. The number of asterisks denote the strength of the difference: * for p values < 0.05; *** for p values <0.001 and a dot instead of an asterisk to denote nearly significant difference. Df, degrees of freedom; F, variance ratio; Pr(>F), P value.
