# Supplementary figures and images for: Soil fauna-microbial interactions shifts fungal and bacterial communities under a contamination disturbance
Source: PLoS One. 2023 Oct 25;18(10):e0292227. doi: 10.1371/journal.pone.0292227 (PMC10599570; doi:10.1371/journal.pone.0292227)

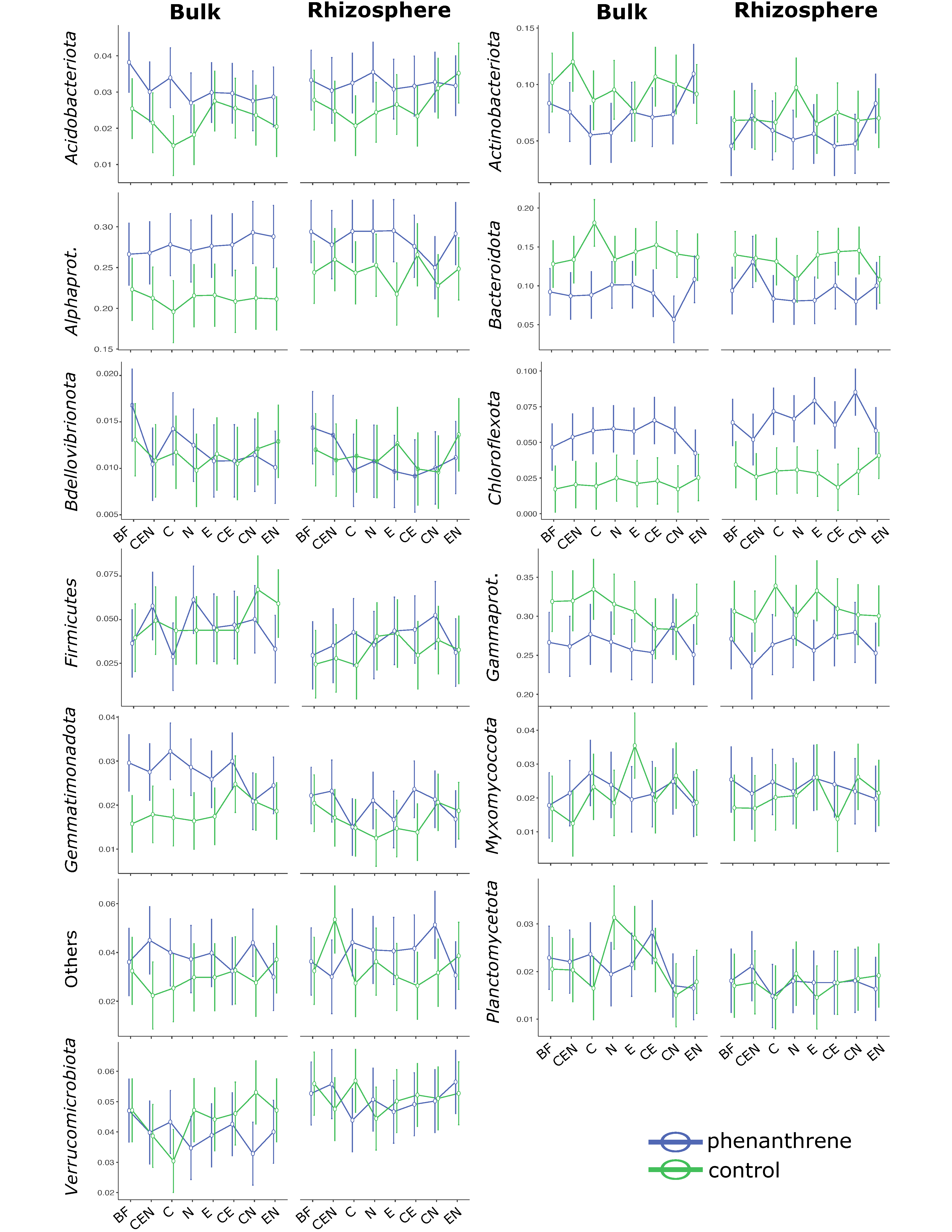

Supplement: S1 Fig — (TIF) [file pone.0292227.s011.tif]
