# Supplementary material for: Soil fauna-microbial interactions shifts fungal and bacterial communities under a contamination disturbance
Source: PLoS One. 2023 Oct 25;18(10):e0292227. doi: 10.1371/journal.pone.0292227 (PMC10599570; doi:10.1371/journal.pone.0292227)
Supplement: Attachment — Submitted filename: Response to Reviewers.docx [file pone.0292227.s012.docx]

**Response to reviewers**

We thank the editor and the reviewers for their thoughtful comments. We have stated our response in blue color under each comment to facilitate the evaluation.

Journal Requirements:

When submitting your revision, we need you to address these additional requirements.

1. Please ensure that your manuscript meets PLOS ONE's style requirements, including those for file naming. The PLOS ONE style templates can be found at

[https://journals.plos.org/plosone/s/file?id=wjVg/PLOSOne_formatting_sample_main_body.pdf](https://can01.safelinks.protection.outlook.com/?url=https%3A%2F%2Fjournals.plos.org%2Fplosone%2Fs%2Ffile%3Fid%3DwjVg%2FPLOSOne_formatting_sample_main_body.pdf&data=05%7C01%7CSara.Garcia%40inrs.ca%7C106b8276015041c367d208dba7ddd7bd%7C80c9267a177f49439958b599ca0e54c2%7C0%7C0%7C638288343464794053%7CUnknown%7CTWFpbGZsb3d8eyJWIjoiMC4wLjAwMDAiLCJQIjoiV2luMzIiLCJBTiI6Ik1haWwiLCJXVCI6Mn0%3D%7C3000%7C%7C%7C&sdata=3WD4xOXENqexFuIifSW3hnh9qxeDAdD70wHL3bjiWx0%3D&reserved=0) and  [https://journals.plos.org/plosone/s/file?id=ba62/PLOSOne_formatting_sample_title_authors_affiliations.pdf](https://can01.safelinks.protection.outlook.com/?url=https%3A%2F%2Fjournals.plos.org%2Fplosone%2Fs%2Ffile%3Fid%3Dba62%2FPLOSOne_formatting_sample_title_authors_affiliations.pdf&data=05%7C01%7CSara.Garcia%40inrs.ca%7C106b8276015041c367d208dba7ddd7bd%7C80c9267a177f49439958b599ca0e54c2%7C0%7C0%7C638288343464794053%7CUnknown%7CTWFpbGZsb3d8eyJWIjoiMC4wLjAwMDAiLCJQIjoiV2luMzIiLCJBTiI6Ik1haWwiLCJXVCI6Mn0%3D%7C3000%7C%7C%7C&sdata=w9I08xrhtOnkjpFeBVLIrwdlSL9atnTp1yF46EMbT5k%3D&reserved=0)

We have now adapted the manuscript to the style requirements.

2. We note that the grant information you provided in the ‘Funding Information’ and ‘Financial Disclosure’ sections do not match.

When you resubmit, please ensure that you provide the correct grant numbers for the awards you received for your study in the ‘Funding Information’ section.

We have now revised the Funding statement.

3. Thank you for stating the following financial disclosure:

"This work was supported by the Natural Sciences and Engineering Research Council of Canada (Discovery grant RGPIN-2014-05274 and strategic grant for projects STPGP 494702) to E.Y S.C.G. was supported by the Research Affiliate Program from the Government of Canada. V.C. and J.A.D. were both supported by the Undergraduate Student Research Awards from the Natural Sciences and Engineering Research Council of Canada. E.M.K. was supported by a scholarship from the Armand-Frappier Foundation. This research was enabled in part by support provided by Calcul Québec ([www.calculquebec.ca](https://can01.safelinks.protection.outlook.com/?url=http%3A%2F%2Fwww.calculquebec.ca%2F&data=05%7C01%7CSara.Garcia%40inrs.ca%7C106b8276015041c367d208dba7ddd7bd%7C80c9267a177f49439958b599ca0e54c2%7C0%7C0%7C638288343464794053%7CUnknown%7CTWFpbGZsb3d8eyJWIjoiMC4wLjAwMDAiLCJQIjoiV2luMzIiLCJBTiI6Ik1haWwiLCJXVCI6Mn0%3D%7C3000%7C%7C%7C&sdata=GhcpGHZDjO%2FYVqu0G2Tsj39by89EUfSXAbqWSPD0qew%3D&reserved=0)) and Compute Canada. "

Please state what role the funders took in the study.  If the funders had no role, please state: "The funders had no role in study design, data collection and analysis, decision to publish, or preparation of the manuscript."  If this statement is not correct you must amend it as needed.

Please include this amended Role of Funder statement in your cover letter; we will change the online submission form on your behalf.

We have now specified the role of the Funder.

4. Thank you for stating the following in the Acknowledgments Section of your manuscript:

"This work was supported by the Natural Sciences and Engineering Research Council of Canada (Discovery grant RGPIN-2014-05274 and strategic grant for projects STPGP 494702) to E.Y S.C.G. was supported by the Research Affiliate Program from the Government of Canada. V.C. and J.A.D. were both supported by the Undergraduate Student Research Awards from the Natural Sciences and Engineering Research Council of Canada. E.M.K. was supported by a scholarship from the Armand-Frappier Foundation. This research was enabled in part by support provided by Calcul Québec ([www.calculquebec.ca](https://can01.safelinks.protection.outlook.com/?url=http%3A%2F%2Fwww.calculquebec.ca%2F&data=05%7C01%7CSara.Garcia%40inrs.ca%7C106b8276015041c367d208dba7ddd7bd%7C80c9267a177f49439958b599ca0e54c2%7C0%7C0%7C638288343464950266%7CUnknown%7CTWFpbGZsb3d8eyJWIjoiMC4wLjAwMDAiLCJQIjoiV2luMzIiLCJBTiI6Ik1haWwiLCJXVCI6Mn0%3D%7C3000%7C%7C%7C&sdata=kCI2unfGb5c%2FDFGN1IYK%2FGG39LBXeox2OObQS7yoSjQ%3D&reserved=0)) and Compute Canada."

We note that you have provided funding information that is not currently declared in your Funding Statement. However, funding information should not appear in the Acknowledgments section or other areas of your manuscript. We will only publish funding information present in the Funding Statement section of the online submission form.

Please remove any funding-related text from the manuscript and let us know how you would like to update your Funding Statement. Currently, your Funding Statement reads as follows:

"This work was supported by the Natural Sciences and Engineering Research Council of Canada (Discovery grant RGPIN-2014-05274 and strategic grant for projects STPGP 494702) to E.Y S.C.G. was supported by the Research Affiliate Program from the Government of Canada. V.C. and J.A.D. were both supported by the Undergraduate Student Research Awards from the Natural Sciences and Engineering Research Council of Canada. E.M.K. was supported by a scholarship from the Armand-Frappier Foundation. This research was enabled in part by support provided by Calcul Québec ([www.calculquebec.ca](https://can01.safelinks.protection.outlook.com/?url=http%3A%2F%2Fwww.calculquebec.ca%2F&data=05%7C01%7CSara.Garcia%40inrs.ca%7C106b8276015041c367d208dba7ddd7bd%7C80c9267a177f49439958b599ca0e54c2%7C0%7C0%7C638288343464950266%7CUnknown%7CTWFpbGZsb3d8eyJWIjoiMC4wLjAwMDAiLCJQIjoiV2luMzIiLCJBTiI6Ik1haWwiLCJXVCI6Mn0%3D%7C3000%7C%7C%7C&sdata=kCI2unfGb5c%2FDFGN1IYK%2FGG39LBXeox2OObQS7yoSjQ%3D&reserved=0)) and Compute Canada."

Please include your amended statements within your cover letter; we will change the online submission form on your behalf.

We have now removed the funding related text from the manuscript

5. In your Data Availability statement, you have not specified where the minimal data set underlying the results described in your manuscript can be found. PLOS defines a study's minimal data set as the underlying data used to reach the conclusions drawn in the manuscript and any additional data required to replicate the reported study findings in their entirety. All PLOS journals require that the minimal data set be made fully available. For more information about our data policy, please see [http://journals.plos.org/plosone/s/data-availability](https://can01.safelinks.protection.outlook.com/?url=http%3A%2F%2Fjournals.plos.org%2Fplosone%2Fs%2Fdata-availability&data=05%7C01%7CSara.Garcia%40inrs.ca%7C106b8276015041c367d208dba7ddd7bd%7C80c9267a177f49439958b599ca0e54c2%7C0%7C0%7C638288343464950266%7CUnknown%7CTWFpbGZsb3d8eyJWIjoiMC4wLjAwMDAiLCJQIjoiV2luMzIiLCJBTiI6Ik1haWwiLCJXVCI6Mn0%3D%7C3000%7C%7C%7C&sdata=mAtUj18xM1gTJ8fx1k5luec3FUM3IDTGaxoOC3jgQWo%3D&reserved=0).

Upon re-submitting your revised manuscript, please upload your study’s minimal underlying data set as either Supporting Information files or to a stable, public repository and include the relevant URLs, DOIs, or accession numbers within your revised cover letter. For a list of acceptable repositories, please see [http://journals.plos.org/plosone/s/data-availability#loc-recommended-repositories](https://can01.safelinks.protection.outlook.com/?url=http%3A%2F%2Fjournals.plos.org%2Fplosone%2Fs%2Fdata-availability%23loc-recommended-repositories&data=05%7C01%7CSara.Garcia%40inrs.ca%7C106b8276015041c367d208dba7ddd7bd%7C80c9267a177f49439958b599ca0e54c2%7C0%7C0%7C638288343464950266%7CUnknown%7CTWFpbGZsb3d8eyJWIjoiMC4wLjAwMDAiLCJQIjoiV2luMzIiLCJBTiI6Ik1haWwiLCJXVCI6Mn0%3D%7C3000%7C%7C%7C&sdata=enmlmKiB0DtEF5v0TC01ZDvlf9ST6t0TYy0L6fDipR0%3D&reserved=0). Any potentially identifying patient information must be fully anonymized.

Important: If there are ethical or legal restrictions to sharing your data publicly, please explain these restrictions in detail. Please see our guidelines for more information on what we consider unacceptable restrictions to publicly sharing data: [http://journals.plos.org/plosone/s/data-availability#loc-unacceptable-data-access-restrictions](https://can01.safelinks.protection.outlook.com/?url=http%3A%2F%2Fjournals.plos.org%2Fplosone%2Fs%2Fdata-availability%23loc-unacceptable-data-access-restrictions&data=05%7C01%7CSara.Garcia%40inrs.ca%7C106b8276015041c367d208dba7ddd7bd%7C80c9267a177f49439958b599ca0e54c2%7C0%7C0%7C638288343464950266%7CUnknown%7CTWFpbGZsb3d8eyJWIjoiMC4wLjAwMDAiLCJQIjoiV2luMzIiLCJBTiI6Ik1haWwiLCJXVCI6Mn0%3D%7C3000%7C%7C%7C&sdata=NuDaJ2MOnzvicy42RdWV2h1XDQQkpsZleR3ffTZfM%2F0%3D&reserved=0). Note that it is not acceptable for the authors to be the sole named individuals responsible for ensuring data access.

We will update your Data Availability statement to reflect the information you provide in your cover letter.

We have now published the minimal underlying data in NCBI (to reproduce the analyses from raw sequences). We have also published the processed count matrices and the data related to the plant, quantitative PCR and the phenanthrene in Zenodo. <https://doi.org/10.5281/zenodo.8299107>

6. We note that you have stated that you will provide repository information for your data at acceptance. Should your manuscript be accepted for publication, we will hold it until you provide the relevant accession numbers or DOIs necessary to access your data. If you wish to make changes to your Data Availability statement, please describe these changes in your cover letter and we will update your Data Availability statement to reflect the information you provide.

Please, see comment above.

7. Please include captions for your Supporting Information files at the end of your manuscript, and update any in-text citations to match accordingly. Please see our Supporting Information guidelines for more information: [http://journals.plos.org/plosone/s/supporting-information](https://can01.safelinks.protection.outlook.com/?url=http%3A%2F%2Fjournals.plos.org%2Fplosone%2Fs%2Fsupporting-information&data=05%7C01%7CSara.Garcia%40inrs.ca%7C106b8276015041c367d208dba7ddd7bd%7C80c9267a177f49439958b599ca0e54c2%7C0%7C0%7C638288343464950266%7CUnknown%7CTWFpbGZsb3d8eyJWIjoiMC4wLjAwMDAiLCJQIjoiV2luMzIiLCJBTiI6Ik1haWwiLCJXVCI6Mn0%3D%7C3000%7C%7C%7C&sdata=sX6P7j6oy7QkXyQMsMenfaME5E1BzKKUwc3260U2Tf0%3D&reserved=0).

We have now included captions for the Supporting information at the end of the manuscript.

8. Please review your reference list to ensure that it is complete and correct. If you have cited papers that have been retracted, please include the rationale for doing so in the manuscript text, or remove these references and replace them with relevant current references. Any changes to the reference list should be mentioned in the rebuttal letter that accompanies your revised manuscript. If you need to cite a retracted article, indicate the article’s retracted status in the References list and also include a citation and full reference for the retraction notice.   [Note: HTML markup is below. Please do not edit.]  Reviewers' comments:  Reviewer's Responses to Questions

We have updated the reference style and revised the reference list.

**Comments to the Author**  1. Is the manuscript technically sound, and do the data support the conclusions?  The manuscript must describe a technically sound piece of scientific research with data that supports the conclusions. Experiments must have been conducted rigorously, with appropriate controls, replication, and sample sizes. The conclusions must be drawn appropriately based on the data presented.

Reviewer #1: Yes

Reviewer #2: Yes

2. Has the statistical analysis been performed appropriately and rigorously?

Reviewer #1: Yes

Reviewer #2: Yes

3. Have the authors made all data underlying the findings in their manuscript fully available?  The [PLOS Data policy](https://can01.safelinks.protection.outlook.com/?url=http%3A%2F%2Fwww.plosone.org%2Fstatic%2Fpolicies.action%23sharing&data=05%7C01%7CSara.Garcia%40inrs.ca%7C106b8276015041c367d208dba7ddd7bd%7C80c9267a177f49439958b599ca0e54c2%7C0%7C0%7C638288343464950266%7CUnknown%7CTWFpbGZsb3d8eyJWIjoiMC4wLjAwMDAiLCJQIjoiV2luMzIiLCJBTiI6Ik1haWwiLCJXVCI6Mn0%3D%7C3000%7C%7C%7C&sdata=7s9IItxqxww2jsktpBjR%2FzWFRB9xQr0oiLDaDq7XwMU%3D&reserved=0) requires authors to make all data underlying the findings described in their manuscript fully available without restriction, with rare exception (please refer to the Data Availability Statement in the manuscript PDF file). The data should be provided as part of the manuscript or its supporting information, or deposited to a public repository. For example, in addition to summary statistics, the data points behind means, medians and variance measures should be available. If there are restrictions on publicly sharing data—e.g. participant privacy or use of data from a third party—those must be specified.

Reviewer #1: Yes

Reviewer #2: Yes

4. Is the manuscript presented in an intelligible fashion and written in standard English?  PLOS ONE does not copyedit accepted manuscripts, so the language in submitted articles must be clear, correct, and unambiguous. Any typographical or grammatical errors should be corrected at revision, so please note any specific errors here.

Reviewer #1: Yes

Reviewer #2: Yes

5. Review Comments to the Author  Please use the space provided to explain your answers to the questions above. You may also include additional comments for the author, including concerns about dual publication, research ethics, or publication ethics. (Please upload your review as an attachment if it exceeds 20,000 characters)

**Reviewer #1:**

Author are recommended to do needful

1.Introduction must be expanded

Done, we have now added a section at the beginning of the introduction where we explore in depth the PAH problematic and phytoremediation as a bioremediation approach suitable for PAH contamination.

2.future prospective must be strengthen

Done. We have now provided both context and future perspectives to the conclusion section.

3.An abstract figure or work flow must be prepared

Done. We have now provided an abstract figure to illustrate the experimental design.

4.RT PCR and Illumina Seq work are remarkable and highly appreciable

Thank you very much!

**Reviewer #2:**

Dear Authors, The manuscript has been written very well and the research plan is very scientific and sound. The MS has shown the original findings and contradictory results with concerned previous studies. It is always good to show the all aspects of research findings either positive or negative.

Thank you very much!

However, it is suggested to revise the title of the manuscript as per finding if possible.

We have now updated the title to be more explicit with the results. Now it reads: Soil fauna-microbial interactions complexity triggers shifts in both fungal and bacterial communities under a contamination disturbance.

Line number 76, potting mix is okay or potting mixture?

Thank you, we have now changed this (L102, 110, 134).

Kindly rewrite the sentence, line number 188-189.

Done, L217 now read as: “Standards were made from 10-fold dilutions of linearized plasmid containing the gene fragment of interest, cloned from soil DNA”

Use brackets to before writing supplementary table name and number in the MS. ex. for line number 201-202

Thank you, we have now corrected this throughout the text to conform to PLOS ONE style.

Kindly check for all the abbreviations used that must be in full form at least ones in the MS.

Done, we have now spelled out all abbreviations on their first mention throughout the manuscript, especially in the material and methods section.

The abstract section will be better if avoid too much abbreviations.

We have now reduced the abbreviations in the abstract.

The conclusion section is too crispy. Therefore, authors can elaborate it in few more lines.

We have now elaborated this section, both in context and perspectives.

The community composition section in the results has been written vastly, authors can reduce these lines by citing concerned tables to increase the readability. For examples, all the species name is not essential to write, it can be referred to the tables.

We have now reduced this part of the results section.

The data analysis and statistical analysis part have been performed very well. The MS can be accepted in this form after suggested few minor corrections.

Thank you very much for your kind comments!

6. PLOS authors have the option to publish the peer review history of their article ([what does this mean?](https://can01.safelinks.protection.outlook.com/?url=https%3A%2F%2Fjournals.plos.org%2Fplosone%2Fs%2Feditorial-and-peer-review-process%23loc-peer-review-history&data=05%7C01%7CSara.Garcia%40inrs.ca%7C106b8276015041c367d208dba7ddd7bd%7C80c9267a177f49439958b599ca0e54c2%7C0%7C0%7C638288343464950266%7CUnknown%7CTWFpbGZsb3d8eyJWIjoiMC4wLjAwMDAiLCJQIjoiV2luMzIiLCJBTiI6Ik1haWwiLCJXVCI6Mn0%3D%7C3000%7C%7C%7C&sdata=dtMcHhVjG1AFhDQ6fDF%2FC04A46ZHDRvRTcSonFKW4Dw%3D&reserved=0)). If published, this will include your full peer review and any attached files.   If you choose “no”, your identity will remain anonymous but your review may still be made public.   **Do you want your identity to be public for this peer review?** For information about this choice, including consent withdrawal, please see our [Privacy Policy](https://can01.safelinks.protection.outlook.com/?url=https%3A%2F%2Fwww.plos.org%2Fprivacy-policy&data=05%7C01%7CSara.Garcia%40inrs.ca%7C106b8276015041c367d208dba7ddd7bd%7C80c9267a177f49439958b599ca0e54c2%7C0%7C0%7C638288343464950266%7CUnknown%7CTWFpbGZsb3d8eyJWIjoiMC4wLjAwMDAiLCJQIjoiV2luMzIiLCJBTiI6Ik1haWwiLCJXVCI6Mn0%3D%7C3000%7C%7C%7C&sdata=Ou6iUBKO38xqJMSNxtFlTjZ%2FLE9MrVfe6qKcSlnMyf0%3D&reserved=0).

Reviewer #1: **Yes:** Dr Kuldip Jayaswall

Reviewer #2: **Yes:** Deepanshu Jayaswal
